# The Retropepsin-Type Protease APRc as a Novel Ig-Binding Protein and Moonlighting Immune Evasion Factor of *Rickettsia*

**DOI:** 10.1128/mBio.03059-21

**Published:** 2021-12-07

**Authors:** Pedro Curto, Andreia Barro, Carla Almeida, Ricardo S. Vieira-Pires, Isaura Simões

**Affiliations:** a CNC - Center for Neuroscience and Cell Biology, University of Coimbra, Coimbra, Portugal; b X-PROT, Cantanhede, Portugal; c III - Institute of Interdisciplinary Research, University of Coimbra, Coimbra, Portugal; Yale University School of Medicine

**Keywords:** *Rickettsia*, APRc, retropepsin, nonimmune immunoglobulin-binding, immune evasion, serum resistance, evasin, aspartic protease

## Abstract

Rickettsiae are obligate intracellular Gram-negative bacteria transmitted by arthropod vectors. Despite their reduced genomes, the function(s) of the majority of rickettsial proteins remains to be uncovered. APRc is a highly conserved retropepsin-type protease, suggested to act as a modulator of other rickettsial surface proteins with a role in adhesion/invasion. However, APRc’s function(s) in bacterial pathogenesis and virulence remains unknown. This study demonstrates that APRc targets host serum components, combining nonimmune immunoglobulin (Ig)-binding activity with resistance to complement-mediated killing. We confirmed nonimmune human IgG binding in extracts of different rickettsial species and intact bacteria. Our results revealed that the soluble domain of APRc is capable of binding to human (h), mouse, and rabbit IgG and different classes of human Ig (IgG, IgM, and IgA) in a concentration-dependent manner. APRc-hIgG interaction was confirmed with total hIgG and normal human serum. APRc-hIgG displayed a binding affinity in the micromolar range. We provided evidence of interaction preferentially through the Fab region and confirmed that binding is independent of catalytic activity. Mapping the APRc region responsible for binding revealed the segment between amino acids 157 and 166 as one of the interacting regions. Furthermore, we demonstrated that expression of the full-length protease in Escherichia coli is sufficient to promote resistance to complement-mediated killing and that interaction with IgG contributes to serum resistance. Our findings position APRc as a novel Ig-binding protein and a novel moonlighting immune evasion factor of *Rickettsia*, contributing to the arsenal of virulence factors utilized by these intracellular pathogens to aid in host colonization.

## INTRODUCTION

Rickettsiae are obligate intracellular bacteria with a limited repertoire of genes, which culminates in a strict dependency on host nutrients and metabolites to survive and proliferate (1). Many rickettsiae are pathogenic to humans, causing infections that range from severe, as in the case of Rocky Mountain spotted fever (Rickettsia rickettsii), Mediterranean spotted fever (Rickettsia conorii), or epidemic typhus (Rickettsia prowazekii), to mild such as those caused by Rickettsia parkeri, Rickettsia africae, and Rickettsia raoultii (2). There is a growing concern about the globally increasing incidence of spotted fever group (SFG) rickettsioses, not only the most severe forms of these diseases but, particularly, milder forms caused by new species of SFG rickettsiae (3). The reemerging character and expanding geographic distribution of SFG *Rickettsia* put humans at substantial risk of exposure and are expected to increase the burden on public health in both developed and developing countries (2, 4). Therefore, gaining a deeper understanding of immune evasion mechanisms and pathogenicity in rickettsiae is fundamental for the development of new approaches to treating rickettsial infections.

Rickettsiae are transmitted by arthropod vectors, and upon inoculation, they require adhesion to and invasion of host cells to establish a successful infection (5, 6). However, before gaining intracellular access, rickettsiae are exposed to complement and antibodies, which are essential features of the host’s innate immunity machinery (7, 8). Several studies have demonstrated that *Rickettsia* bacteria are resistant to serum bactericidal effects and can evade complement-mediated killing (9–13). Thus far, three rickettsial surface proteins have been identified as important contributors to serum resistance (10, 12, 13): the rickettsial autotransporter protein rOmpB, which specifically interacts with factor H (a soluble host complement inhibitor) (12), and the rickettsial outer membrane proteins Adr1 and Adr2, which interact with the terminal complement complex inhibitor vitronectin (10, 13). These protein factors illustrate two different mechanisms mediating partial survival of *Rickettsia* in human serum through recruitment of regulators of complement activation (14), clearly suggesting that rickettsial species may have evolved multiple mechanisms to inhibit recognition by host serum components.

The significance of the host’s innate surveillance mechanisms for pathogen clearance is demonstrated by the diverse arsenal of virulence determinants and evasive strategies identified in many human-pathogenic bacteria capable of interfering with complement and, thereby, promoting immune evasion (7, 8). Bacterial surface proteins capable of nonimmune immunoglobulin (Ig) binding are key players in immune evasion due to protection against complement attack, decreasing opsonization and phagocytosis, or both (15–17). Moreover, a second known function of proteins binding to Ig, without involving the antigen-binding site, is to act as B-cell or immunoglobulin superantigens (18–20). Therefore, Ig-binding proteins are considered important factors of pathogenicity, although, in general, there is still a limited understanding of their role in virulence. There are many Ig-binding proteins, differing significantly in structure, size, binding properties/affinities, and binding sites on the Ig molecules (15, 17). The best characterized are from Gram-positive bacteria and include protein A (SpA), Sbi, IsaB, and SSL10 from Staphylococcus aureus (15, 17, 21–23), protein M and M-like proteins from group A streptococci (16), protein G from group C and G streptococci (17, 24), and protein L from Finegoldia magna (25). Although not as well explored, it has been demonstrated that Gram-negative bacteria also express nonimmune Ig-binding proteins. They have been found in Histophilus somni (26); within the pathogenic *Yersinia* genus, Y. pestis and Y. pseudotuberculosis (27–29); Stenotrophomonas maltophilia (30); Escherichia coli (31); different species of *Mycoplasma* (32–34); and Helicobacter pylori (35). Importantly, many nonimmune Ig-binding proteins are known to be multifunctional, interacting with other host serum proteins or immune cells, which further contributes to providing resistance against clearance by the host’s innate and adaptive immune systems (16, 17).

APRc is a rickettsial membrane-embedded retropepsin-type aspartic protease previously identified in our laboratory (36), whose gene is conserved in all 100 sequenced *Rickettsia* genomes. Using the R. conorii gene homolog as a working model, we have shown that APRc shares common properties with viral retropepsins, including autolytic activity, activity dependent on dimer formation, optimum pH, specificity preferences, and partial inhibition by HIV-1 protease inhibitors (36). Moreover, structural results unequivocally demonstrated that the APRc monomer follows the canonical fold observed in all retropepsins, either of viral or of eukaryotic origin (37). The unique features of APRc—accumulation in rickettsial outer membrane with a catalytic domain extracellularly oriented, the observable nonstringent sequence specificity, and the autoprocessing activity that may indicate a release of the soluble catalytic domain of APRc from the surface of the cells by an ectodomain shedding-like process (36)—combined with its strict conservation in rickettsial genomes anticipate a potential multifunctional role for this protease in the rickettsial life cycle. While, for other classes of proteases, their role in bacterial pathogenesis and virulence has been extensively explored (38, 39), virtually nothing is known about the role of aspartic proteases in these processes since their presence in pathogenic bacteria has only recently been acknowledged (36 and our unpublished data). We have previously demonstrated *in vitro* processing of two conserved autotransporter proteins, Sca5/OmpB and Sca0/OmpA, by APRc, suggesting its potential role as a modulator of other rickettsial surface proteins involved in adhesion to and invasion of host cells (36).

In this work, we provide evidence that APRc also targets host cell components, displaying nonimmune immunoglobulin (Ig)-binding activity and participating in serum resistance. Together, our findings anticipate APRc as a novel moonlighting protein, acting as a novel evasin contributing to *Rickettsia*’s immune evasion toolbox.

## RESULTS

### Immunoglobulin G binds at the surface of *Rickettsia* species.

**FIG 1:**
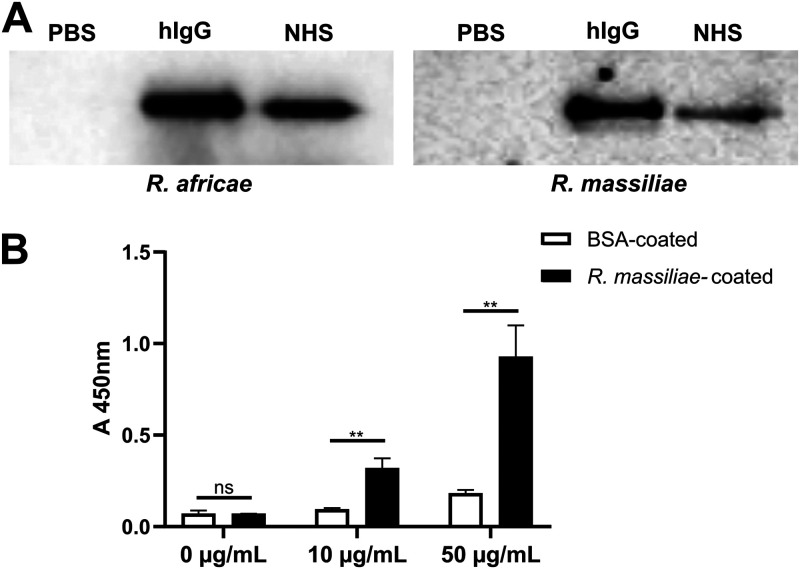
Nonimmune IgG binding at the surface of *Rickettsia* species. (A) PFA-fixed *Rickettsia* (*R. africae* and *R. massiliae*) bacteria were incubated with human IgG (hIgG), 50% NHS, or PBS as control for 2 h at 37°C. After incubation, bacteria were washed twice with PBS, and proteins that bound at the surface of *Rickettsia* were then eluted with PBS containing 1 M NaCl and analyzed by Western blotting with HRP-labeled anti-human IgG Fc antibody. (B) PFA-fixed *R. massiliae* bacteria were applied as a coating onto 96-well plates at 4.5 × 10^7^ bacteria per well. BSA at 1 μg/well was used as a negative control. Nonimmune IgG binding was then evaluated by incubation with HRP-labeled rabbit IgG at different concentrations. Binding was detected at 450 nm. Data represent mean values ± standard deviations from three replicates. Significance was determined by an unpaired *t* test using GraphPad Prism 8 (ns, not significant; **, *P* < 0.01).

Several successful pathogenic bacteria express proteins at their surface with the ability to bind immunoglobulins without the requirement of antigen-binding sites as a possible mechanism to protect bacteria from the action of the complement system (17). To evaluate if *Rickettsia* species have the capacity to bind IgG at their surface, we have incubated paraformaldehyde (PFA)-fixed *Rickettsia* species (*R. africae* and R. massiliae) with human IgG, normal human serum (NHS), or phosphate-buffered saline (PBS) as a control. Bacterium-bound proteins were eluted with salt and analyzed by Western blotting with an anti-human IgG Fc antibody. Our results demonstrate the association of IgG at the surface of both rickettsial species, when using isolated human IgG or in the context of a more complex mixture as NHS (Fig. 1A). We have further validated these results by whole-cell enzyme-linked immunosorbent assay (ELISA). To this end, PFA-fixed *R. massiliae*, or bovine serum albumin (BSA) as a negative control, was applied as a coating onto 96-well plates and evaluated for its ability to bind horseradish peroxidase (HRP)-labeled rabbit IgG. Again, our results demonstrate the specific association of IgG at the surface of *Rickettsia* in a concentration-dependent manner (Fig. 1B).

### The rickettsial retropepsin APRc is a potential nonimmune IgG-binding protein.

**FIG 2:**
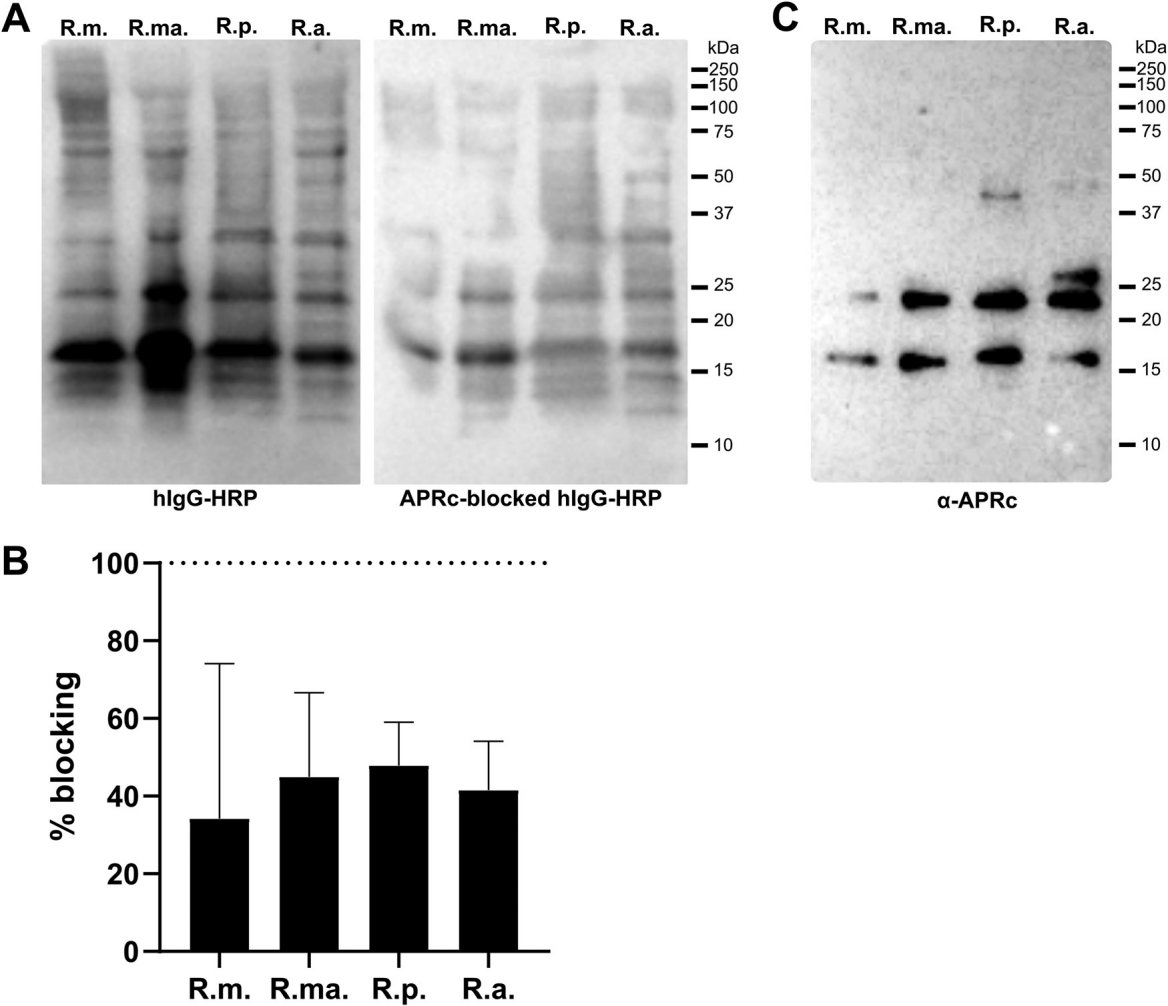
APRc as a potential nonimmune IgG-binding protein at the surface of *Rickettsia*. (A) Total protein extracts (12 μg) from different *Rickettsia* species (R. montanensis [R.m.], *R. massiliae* [R.ma.], *R. parkeri* [R.p.], and *R. africae* [R.a.]) were assayed for the presence of nonimmune IgG-binding proteins. Rickettsial protein extracts were evaluated by far-Western blotting with HRP-labeled human IgG (left) or HRP-labeled human IgG previously blocked with purified recombinant soluble domain APRc (APRc_110–231_His) catalytically inactive at a ratio of 1:550 m/m (hIgG to APRc) for 3 h at room temperature (right). (B) Quantification of far-Western blotting for the protein bands with molecular weights of approximately 24 and 16 kDa in rickettsial extracts probed with HRP-labeled human IgG versus APRc-blocked HRP-labeled human IgG. Percentage of blocking was determined as follows: 100 − [(band intensity for APRc-blocked HRP-labeled human IgG/band intensity for HRP-labeled human IgG) × 100]. Data represent mean values ± standard deviations from three independent replicates. (C) Total protein extracts (12 μg) from different *Rickettsia* species (R. montanensis [R.m.], *R. massiliae* [R.ma.], *R. parkeri* [R.p.], and *R. africae* [R.a.]) were evaluated by Western blotting with an anti-APRc antibody.

To search for candidate nonimmune IgG-binding proteins in rickettsiae, total protein extracts from Rickettsia montanensis, *R. massiliae*, *R. parkeri*, and *R. africae* were separated by SDS-PAGE, transferred to a polyvinylidene difluoride (PVDF) membrane, and probed with an HRP-labeled human IgG antibody. Our results show human IgG interaction with two protein bands with molecular weights of approximately 24 and 16 kDa in all rickettsial extracts (Fig. 2A, left), further corroborating the presence of IgG-binding proteins in *Rickettsia*. Interestingly, these protein bands correspond to similar molecular weights of the multistep processing forms of the retropepsin-like protease APRc, the outer membrane protein of rickettsiae previously characterized in our laboratory (36, 37). Therefore, we sought to evaluate if APRc could be the protein responsible for the nonimmune IgG-binding activity. To assess that, we performed a parallel competition assay where total protein extracts from the different rickettsial species were probed with HRP-labeled human IgG antibody previously blocked with a catalytic inactive recombinant purified form of APRc soluble domain (APRc_110–231_His) (see Fig. S1G in the supplemental material). As shown in Fig. 2A (right) and Fig. 2B, incubation of APRc with human IgG impaired the nonimmune IgG binding observed in total rickettsial extracts, anticipating APRc as a potential candidate protein for this activity. The specific detection of native APRc products at similar molecular weights (∼24 and 16 kDa) in these extracts is shown for comparison (Fig. 2C).

10.1128/mBio.03059-21.1 FIG S1 Purification of APRc_110–231_His and APRc_100–231_(D140N)His by immobilized-metal affinity chromatography (IMAC) Ni^2+^ and cation-exchange chromatography. (A to D) Purification of APRc_110–231_His. (A) The recombinant protein APRc_110–231_His was purified by IMAC Ni^2+^ on a HisTrap HP 5-ml column, previously equilibrated in 20 mM sodium phosphate (pH 7.4) buffer, containing 10 mM imidazole and 500 mM NaCl. Protein elution was carried out by implementation of a four-step gradient of imidazole (50 mM, 150 mM, 200 mM, and 500 mM) in 20 mM sodium phosphate (pH 7.4) buffer containing 500 mM NaCl (represented by the gray dashed line) at a flow rate of 5 ml/min and monitored at an absorbance of 280 nm. The eluted proteins of the 200 mM step (pool 2) were dialyzed overnight against 20 mM HEPES (pH 7.4) buffer. (B) After dialysis, samples from pool 2 were purified in a Mono-S 5/50 GL column, previously equilibrated with 20 mM HEPES (pH 7.4) buffer. Protein elution was carried out by implementation of a linear gradient of NaCl (0 to 1 M) in 20 mM HEPES (pH 7.4) buffer, at a flow rate of 0.75 ml/min and monitored at an absorbance of 280 nm. The conductivity is represented by the gray dashed line. The selected eluted fraction (2) is outlined by dashed lines. (C) SDS-PAGE analysis followed by Coomassie blue staining of the eluted fraction (2) from the purification by cation-exchange chromatography. Twenty microliters of sample was denatured with SDS loading buffer and then applied to a 12.5% polyacrylamide gel. (D to F) Purification of APRc_100–231_(D140N)His. (D) The recombinant protein APRc_110–231_(D140N)His was purified by IMAC Ni^2+^ on a HisTrap HP 5-ml column under the same conditions as described for panel A. The eluted fractions of the 200 mM imidazole gradient step were pooled (pool 1) and dialyzed overnight against 20 mM HEPES (pH 7.4) buffer. (E) After dialysis, the protein was purified in a Mono-S 5/50 GL column, under the same conditions as described for panel B. The selected eluted fraction (1) is outlined by dashed lines. (F) SDS-PAGE analysis followed by Coomassie blue staining of the pooled eluted fraction resulting from the purification by cation-exchange chromatography. Twenty microliters of sample was denatured with SDS loading buffer and then applied to a 12.5% polyacrylamide gel. (G and H) Samples of the eluted fractions of recombinant dimeric APRc_110–231_His WT and mutant and monomeric WT were incubated with oxidized insulin β-chain overnight at 37°C, in 0.1 M sodium acetate buffer, pH 6. Proteolytic activity was then assessed by reversed-phase HPLC (RP-HPLC). As a negative control, oxidized insulin β-chain was incubated with sample buffer. The elution of peptides was carried out with a linear gradient of acetonitrile (0 to 80%) in 0.1% trifluoroacetic acid (TFA), at a flow rate of 1 ml/min, and monitored at an absorbance of 220 nm. The peaks corresponding to the peptides originating from the digestion of oxidized insulin β-chain are marked with an asterisk. Download FIG S1, TIF file, 1.2 MB. Copyright © 2021 Curto et al. 2021 Curto et al. https://creativecommons.org/licenses/by/4.0/ This content is distributed under the terms of the Creative Commons Attribution 4.0 International license.

### APRc binds IgGs from different origins as well as different classes of immunoglobulins.

**FIG 3:**
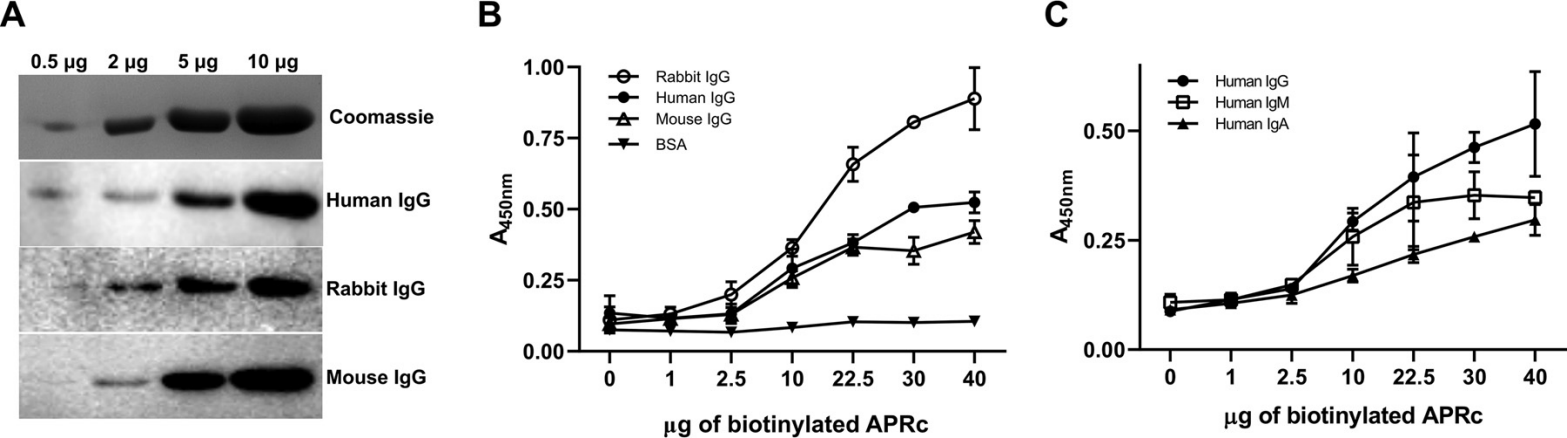
APRc binds IgGs from different origins as well as different classes of Igs. (A) Different amounts of recombinant and purified APRc were assessed for their ability to bind IgGs from different mammalian origins (human IgG, rabbit IgG, and mouse IgG) by far-Western blotting. (B) The ability of APRc to bind IgGs from different origins was evaluated by ELISA. BSA and IgGs from rabbit IgG, human IgG, and mouse IgG were applied as a coating onto 96-well plates at 1 μg/well and incubated with different concentrations of biotinylated APRc. Binding was then detected with HRP-conjugated streptavidin at 450 nm. Data represent mean values ± standard deviations from three replicates. (C) Binding of APRc to different classes of immunoglobulins was assessed by ELISA. Human immunoglobulins from different classes (IgG, IgM, and IgA) were applied as a coating onto 96-well plates at 1 μg/well and incubated with different concentrations of biotinylated APRc. Binding was then detected with HRP-conjugated streptavidin at 450 nm. Data represent mean values ± standard deviations from three replicates.

To confirm the nonimmune IgG-binding activity of APRc, we evaluated the ability of different amounts of its purified recombinant untagged soluble domain (APRc_110–231_) (Fig. S2) to bind IgGs from different mammalian origins by far-Western blotting (Fig. 3A). Our results demonstrate that APRc can bind human (h), rabbit (r), and mouse (m) IgGs in a concentration-dependent manner. We have further confirmed these results by ELISA. To this end, we have recombinantly expressed and purified a biotinylated form of the soluble domain of APRc (Fig. S3). The ability of biotinylated APRc to bind human, rabbit, and mouse IgG was then evaluated with streptavidin-HRP detection. Again, our data demonstrate that APRc can bind IgGs of several mammalian origins in a concentration-dependent manner (rIgG > hIgG > mIgG) whereas no binding is observed for BSA-coated wells used as a negative control (Fig. 3B). We next evaluated if APRc could also bind different classes of human immunoglobulins (Igs). We have assayed the capacity of biotinylated APRc to bind to human IgG, IgM, and IgA. As shown in Fig. 3C, our results confirm that APRc can bind to all tested classes of human Igs, displaying higher interaction with IgG. As an additional control, this nonimmune IgG-binding capacity of APRc was further confirmed by ELISA with a mouse monoclonal antibody against an unrelated target (Fig. S4).

10.1128/mBio.03059-21.2 FIG S2 Purification of the untagged version of the soluble domain of APRc_110–231_. (A) The construct pCoofy1_APRc_110–231_ was purified by IMAC Ni^2+^ on a HisTrap HP 5-ml column, previously equilibrated in 20 mM sodium phosphate (pH 7.4) buffer, containing 10 mM imidazole and 500 mM NaCl. Protein elution was carried out by implementation of a four-step gradient of imidazole (50 mM, 150 mM, 200 mM, and 500 mM) in 20 mM sodium phosphate (pH 7.4) buffer containing 500 mM NaCl (represented by the gray dashed line) at a flow rate of 5 ml/min and monitored at an absorbance of 280 nm. The eluted proteins of the 150 mM and 500 mM imidazole gradient steps were pooled and dialyzed overnight against PBS, pH 7.4. (B) After dialysis, samples were applied onto a HiLoad 26/60 Superdex 200 gel filtration column, previously equilibrated in PBS (pH 7.4) at a flow rate of 2.0 ml/min. The protein fractions were further concentrated and incubated with HRV 3C protease in an m/m ratio of 1:33 (HRV 3C to APRc) in 50 mM Tris-HCl (pH 7.5) containing 150 mM NaCl at 4°C for N terminus hexahistidine tag removal. After digestion, samples were incubated with Ni Sepharose high-performance beads for 15 min at room temperature with agitation, filtered through 0.2-μm filters, and dialyzed against PBS, pH 7.4. (C) SDS-PAGE analysis followed by Coomassie blue staining of protein fractions eluted from a HiLoad 26/60 Superdex 200 gel filtration column (1), after incubation with HRV 3C protease (2), and after the cleanup step with Ni Sepharose beads, filtration, and dialysis against PBS pH 7.4 (3). Five micrograms of each sample was denatured with SDS loading buffer and then applied to a 12.5% polyacrylamide gel. Download FIG S2, TIF file, 0.4 MB. Copyright © 2021 Curto et al. 2021 Curto et al. https://creativecommons.org/licenses/by/4.0/ This content is distributed under the terms of the Creative Commons Attribution 4.0 International license.

10.1128/mBio.03059-21.3 FIG S3 Production of the biotinylated form of APRc soluble domain. (A) The recombinant biotinylated form of the APRc soluble domain was purified by IMAC Ni^2+^ on a HisTrap HP 5-ml column, previously equilibrated in 20 mM sodium phosphate (pH 7.4) buffer, containing 10 mM imidazole and 500 mM NaCl. Protein elution was carried out by a four-step gradient of imidazole (50 mM, 150 mM, 200 mM, and 500 mM) in 20 mM sodium phosphate (pH 7.4) buffer containing 500 mM NaCl (represented by the gray dashed line) at a flow rate of 5 ml/min and monitored at an absorbance of 280 nm. The eluted fractions of the 150 mM imidazole gradient step were pooled (pool 1) and dialyzed overnight against 20 mM Tris-HCl (pH 8) buffer. (B) After dialysis, the protein was purified by anion-exchange chromatography in a Mono-Q 5/50 GL column, previously equilibrated with 20 mM Tris-HCl, pH 8. Protein elution was carried out by a linear gradient of NaCl (0 to 1 M) in 20 mM Tris-HCl (pH 8) buffer at a flow rate of 0.75 ml/min and monitored at an absorbance of 280 nm. The conductivity (represented by the gray dashed line) was also monitored. The selected eluted fractions (1 and 2) are outlined by dashed lines. (C) SDS-PAGE analysis followed by Coomassie blue staining of the selected eluted fractions 1 and 2 from the purification by anion-exchange chromatography. Twenty microliters of each sample was denatured with SDS loading buffer with DTT and then applied to a 12.5% polyacrylamide gel. (D) His tag removal from biotinylated APRc. The collected fractions 1 and 2 of biotinylated APRc were incubated with HRV 3C protease in 50 mM Tris-HCl (pH 7.5) buffer containing 150 mM NaCl overnight at 4°C followed by incubation with Ni Sepharose high-performance beads for 15 min at room temperature. The Ni Sepharose high-performance beads were removed, and a dialysis was performed against PBS for 3 h at 4°C. Samples from before (T_0_) and after (T_f_) digestion and after purification with Ni Sepharose high-performance beads were collected and analyzed by SDS-PAGE followed by Coomassie blue staining. Five microliters of each sample was applied in a 12.5% polyacrylamide gel. (E) Confirmation of binding between biotinylated APRc and human IgG. Ninety-six-well ELISA plates were coated with 1 μg of human IgG (catalog no. I2511; Sigma-Aldrich) per well followed by incubation at 37°C for 2 h. The remaining protein-binding sites in the coated wells were blocked by incubation at 37°C for 2 h with PBS-T buffer containing 3% BSA and then incubated with different amounts of biotinylated APRc (0 μg/well, 1 μg/well, 2.5 μg/well, 10 μg/well, and 22.5 μg/well) diluted in PBS, followed by incubation at 37°C for 1 h. HRP-conjugated streptavidin (Cell Signaling Technology) (1:2,000) in PBS-T buffer containing 3% milk was added to each well and incubated at 37°C for 1 h. The 1-Strep Ultra TMB ELISA substrate was added to each well and incubated at room temperature for 15/20 min followed by the addition of 2 M sulfuric acid per well. The evaluation of biotinylated APRc binding to human IgG was performed through detection of HRP activity toward the TMB substrate at an absorbance of 450 nm. Download FIG S3, TIF file, 1.9 MB. Copyright © 2021 Curto et al. 2021 Curto et al. https://creativecommons.org/licenses/by/4.0/ This content is distributed under the terms of the Creative Commons Attribution 4.0 International license.

10.1128/mBio.03059-21.4 FIG S4 APRc binds to a mouse monoclonal antibody targeting an unrelated protein. The ability of APRc to bind to a monoclonal antibody was evaluated by ELISA. BSA and mouse monoclonal IgG1 were applied as a coating onto 96-well plates at 1 μg/well and incubated with different concentrations of biotinylated APRc. Binding was then detected with HRP-conjugated streptavidin at 450 nm. Data represent mean values ± standard deviations from three replicates. Download FIG S4, TIF file, 0.10 MB. Copyright © 2021 Curto et al. 2021 Curto et al. https://creativecommons.org/licenses/by/4.0/ This content is distributed under the terms of the Creative Commons Attribution 4.0 International license.

### APRc-hIgG binding stabilizes the APRc oligomeric state but does not entail IgG cleavage.

**FIG 4:**
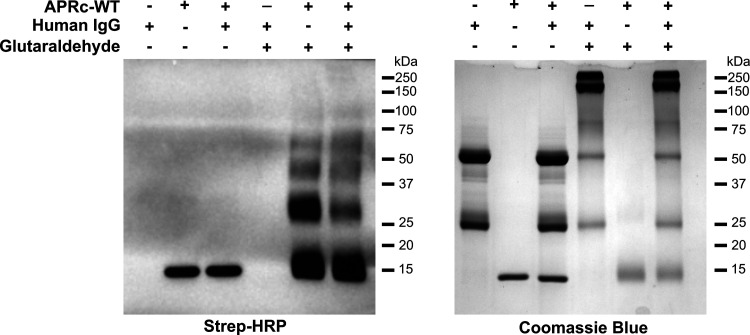
APRc-hIgG binding stabilizes the APRc oligomeric state. Recombinant biotinylated APRc_110–231_ was incubated with human IgG at equimolar ratios (166.85 pmol) in PBS, pH 7.4, for 4 h at 37°C. After incubation, samples were treated with glutaraldehyde, or water as a control, and incubated for 4 min at 37°C. The reaction was stopped by adding 1 M Tris-HCl, pH 8.0, and the products of incubation were resolved by SDS-PAGE under reducing conditions and assessed by Western blotting with HRP-streptavidin (left) and Coomassie blue staining as evidence for loading (right). WT, wild type.

For additional evaluation of APRc-IgG binding, equimolar ratios of biotinylated APRc and human IgG were incubated, and at the end of the incubation, samples were treated with glutaraldehyde (or water, as a negative control for the cross-linking). Reaction products and control samples were denatured under reducing conditions and then analyzed by Western blotting with streptavidin-HRP and Coomassie blue staining as evidence for loading (Fig. 4). Our results demonstrate that there is a shift in the signal corresponding to the dimeric form of APRc (∼28 kDa), upon incubation with IgG, with an increment in signal for higher molecular weights.

There are several examples of microbial proteases that degrade human Igs, thereby contributing to bacterial evasion of Ig functions (15). Since APRc is an active aspartic protease (36), we also aimed to determine if incubation of active recombinant APRc and its catalytic mutant (APRcD140N) with human IgG would result in alterations in migration profiles of either interacting partner. Thus, we independently incubated the recombinant soluble domain of wild-type (APRc_110–231_His) and mutant [APRc_110–231_(D140N)His] APRc with human IgG at equimolar ratios and assessed changes in migration patterns by Western blotting after resolving the incubation products under nonreducing conditions. Controls with only human IgG or wild-type and mutant APRc incubated under the same conditions were performed in parallel (Fig. S5A). Our results show again that, in the presence of IgG, the dimeric form of APRc (∼28 kDa) undergoes a shift to higher molecular weights observable for both wild-type and mutant forms. These observations further confirm the interaction of APRc with human IgG, suggesting that APRc-hIgG binding may stabilize the oligomeric state of the protease (as shown in Fig. 4). Moreover, these results demonstrate that this interaction is independent of APRc catalytic activity. From the Western blot assay with anti-human IgG antibody (also under nonreducing conditions [Fig. S5B]) (and under reducing conditions [data not shown]), no apparent differences were observed on IgG between incubations with APRc wild-type and mutant forms. Several incubation conditions were additionally tested (different enzyme:substrate [E:S] ratios, pHs, incubation times), but no evidence for IgG cleavage products was ever observed (data not shown). Therefore, our results suggest that APRc-hIgG binding does not entail the cleavage of the antibody.

10.1128/mBio.03059-21.5 FIG S5 APRc-hIgG binding stabilizes the APRc oligomeric state but does not entail IgG cleavage. (A and B) Recombinant soluble domains of wild-type (APRc-WT) and mutant (APRc-D140N) forms of APRc_110–231_His were incubated with human IgG at equimolar ratios (333.7 pmol) in PBS (pH 7.4) for 4 h at 37°C. Changes in migration patterns were assessed by Western blotting with anti-APRc antibody (A) and anti-human antibody (B) after resolving the incubation products by SDS-PAGE under nonreducing conditions. Download FIG S5, TIF file, 0.6 MB. Copyright © 2021 Curto et al. 2021 Curto et al. https://creativecommons.org/licenses/by/4.0/ This content is distributed under the terms of the Creative Commons Attribution 4.0 International license.

### APRc binds to human IgG preferentially through the Fab region.

**FIG 5:**
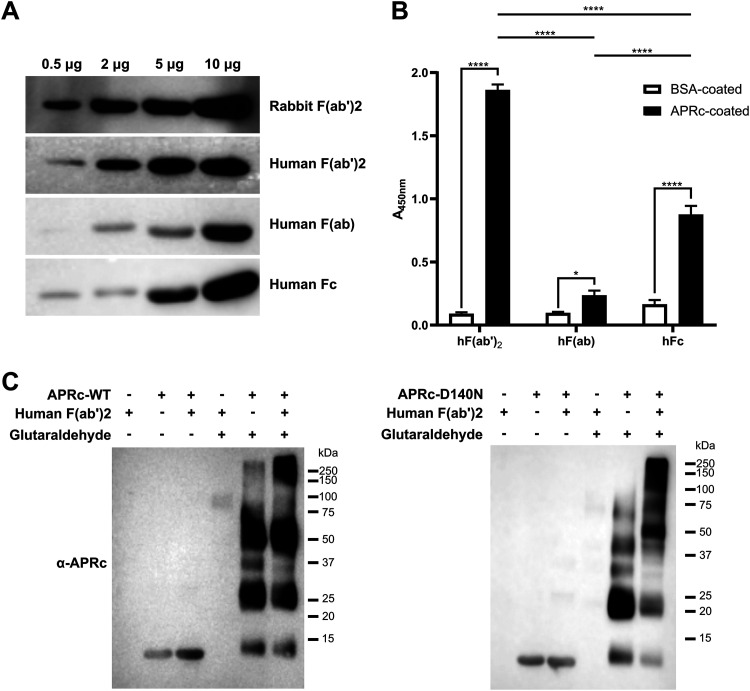
APRc binds to IgG preferentially through the Fab region. (A) Different amounts of recombinant soluble domain of APRc were assessed for their ability to bind rabbit HRP-F(ab′)_2_ and human HRP-F(ab′)_2_, HRP-F(ab′), and HRP-Fc domain by far-Western blotting. (B) The ability of APRc to bind to the different human IgG fragments was evaluated by ELISA. BSA and APRc_110–231_His were applied as a coating onto 96-well plates at 1 μg/well and incubated with 9.09 pmol/well of HRP-labeled human F(ab′)_2_, F(ab′), and Fc. Binding was detected upon incubation with HRP substrate at 450 nm. Data represent mean values ± standard deviations from three replicates. Significance was determined by two-way ANOVA followed by Tukey multiple-comparison test using GraphPad Prism 8 (*, *P* < 0.05; ****, *P* < 0.0001). (C) Differences in migration of APRc wild type (WT) and mutant in the presence of human F(ab′)_2_. Recombinant soluble domains of wild-type (APRc-WT; left panel) and mutant (APRc-D140N; right panel) APRc_110–231_His were incubated with human F(ab′)_2_ at an equimolar ratio (166.85 pmol), for 4 h at 37°C. After incubation, samples were treated with glutaraldehyde, or water as a control, and incubated for 4 min at 37°C. The reaction was stopped by adding 1 M Tris-HCl, pH 8.0, and the products of incubation were resolved by SDS-PAGE under reducing conditions and assessed by Western blotting with an anti-APRc antibody.

For the evaluation of the region in IgG responsible for binding to APRc, we produced F(ab′)_2_ and F(ab′) (Fig. S6) or used commercially available versions of these fragments as well as of human Fc. We started assessing the nonimmune binding activity of APRc to IgG fragments by far-Western blotting (Fig. 5A). F(ab′)_2_ fragments from human (h) and rabbit (r) IgGs, h F(ab′), and h Fc, labeled with HRP, were used to probe binding to different amounts of the soluble domain of APRc. As shown in Fig. 5A, APRc binds more strongly to h/r F(ab′)_2_ in a concentration-dependent manner. As an independent validation of these results, we have performed an ELISA providing the relative quantification of APRc binding to these human IgG fragments. Purified recombinant APRc (APRc_110–231_His) and BSA as a negative control were applied as a coating onto 96-well plates and evaluated for their ability to bind HRP-labeled h F(ab′)_2_, h F(ab′), and h Fc. As shown in Fig. 5B, APRc displays a significantly higher binding capacity to F(ab′)_2_ compared with Fc or the monovalent fragment F(ab′). These results confirm the previous observations by far-Western blotting of APRc binding to IgG preferentially through the Fab region. To further ensure if this interaction is independent of APRc’s catalytic activity, we have performed cross-linking assays with recombinant dimeric APRc_110–231_His and APRc_110–231_(D140N)His. F(ab′)_2_ fragments were independently incubated with both forms of the protease at equimolar ratios, and at the end of the incubation, samples were treated with glutaraldehyde (or water). Reaction products and control samples were then analyzed by Western blotting with an anti-APRc antibody (Fig. 5C; see also Fig. S7, as loading control). As shown in Fig. 5C, for both cross-linking incubations with the wild-type and mutant forms of APRc, we observe evident differences in APRc migration in the presence of human F(ab′)_2_, with a shift of the protease signal to higher molecular weights. These results confirm again that the interaction is independent of the catalytic activity of APRc.

10.1128/mBio.03059-21.6 FIG S6 Production and purification of human F(ab′)_2_ and F(ab′) fragments. (A to C) F(ab′)_2_ production and purification from the digestion of human IgG with pepsin. (A) Human IgG (catalog no. I2511; Sigma-Aldrich) was digested with pepsin in 50 mM sodium acetate (pH 4.0) buffer containing 100 mM NaCl, for 8 h 30 min at 37°C. Samples from before (T_0_) and after (T_f_) digestion with pepsin were collected, denatured with SDS loading buffer (with and without DTT), and evaluated by SDS-PAGE followed by Coomassie blue staining. Ten microliters of each sample was applied to a 10% polyacrylamide gel. (B) After confirmation of the digestion of the human IgG, 10 μM pepstatin was added to stop the digestion, and the sample was diluted in PBS buffer and further concentrated in a Vivaspin 500, 50-kDa PES column. Samples were collected after concentration and denatured with SDS loading buffer with and without DTT. Three microliters of each sample was applied in a 10% polyacrylamide gel and then evaluated by Western blot analysis with the monoclonal mouse anti-human IgG Fc (HRP) (A01854-200; GenScript) (1:20,000 in TBS-T, 2% BSA). (C) To remove the Fc fragments resulting from the digestion of IgG with pepsin as well as the undigested human IgG, the sample was incubated with protein A Mag Sepharose at room temperature for 2 h. After the incubation, the resin was separated and the supernatant containing F(ab′)_2_ was collected. Intact human IgG and F(ab′)_2_ preparations (0.5 μg) were then analyzed by Western blotting with the polyclonal rabbit anti-mouse IgG (whole molecule)-peroxidase (A9044; Sigma-Aldrich) (1:5,000 in TBS-T, 2% BSA) for quality control. (D and E) F(ab′) production and purification from the digestion of human F(ab′)_2_ with papain. (D) Human F(ab′)_2_ (pepsin-digested samples containing Fc fragments) was digested with papain in PBS (pH 7.4) buffer containing 0.02 M EDTA and 0.02 M cysteine for 1 h 30 min at 37°C. Samples from before (T_0_) and after (T_f_) digestion with papain were collected, denatured with SDS loading buffer (with and without DTT), and evaluated by SDS-PAGE followed by Coomassie blue staining. Ten microliters of each sample was applied to a 10% polyacrylamide gel. (E) After confirmation of the digestion, sample was diluted in 20 ml of PBS (pH 7.4) buffer and concentrated in an Amicon Ultra-4 centrifugal filter unit (Ultracel 3,000 Da). To remove the Fc fragments, the sample was further incubated with protein A Mag Sepharose at room temperature for 2 h. After the incubation, the resin was separated and the supernatant containing F(ab′) was collected. To stop the digestion, 10 μM E-64 protease inhibitor was added. For quality control, intact human IgG and F(ab′) preparations (0.5 μg) were then analyzed by Western blotting with the monoclonal mouse anti-human Ig kappa light chain (MAB10050; R&D Systems, Bio-techne) (1:259 in TBS-T, 2% BSA) followed by the polyclonal rabbit anti-mouse IgG (whole molecule)-peroxidase (A9044; Sigma-Aldrich) (1:10,000 in TBS-T, 2% BSA) or with the monoclonal mouse anti-human IgG Fc (HRP) (A01854-200; GenScript) (1:20,000 in TBS-T, 2% BSA). Download FIG S6, TIF file, 1.3 MB. Copyright © 2021 Curto et al. 2021 Curto et al. https://creativecommons.org/licenses/by/4.0/ This content is distributed under the terms of the Creative Commons Attribution 4.0 International license.

10.1128/mBio.03059-21.7 FIG S7 Differences in migration of APRc WT in the presence of human F(ab′)_2_. Recombinant soluble domain of wild-type APRc_110–231_His (APRc-WT) was incubated with human F(ab′)_2_ at an equimolar ratio (166.85 pmol), for 4 h at 37°C. After incubation, samples were treated with glutaraldehyde, or water as a control, and incubated for 4 min at 37°C. The reaction was stopped, and the products were resolved by SDS-PAGE under reducing conditions and assessed by Coomassie blue staining as evidence for loading. Download FIG S7, TIF file, 0.5 MB. Copyright © 2021 Curto et al. 2021 Curto et al. https://creativecommons.org/licenses/by/4.0/ This content is distributed under the terms of the Creative Commons Attribution 4.0 International license.

### Binding affinity between APRc and human IgG.

**FIG 6:**
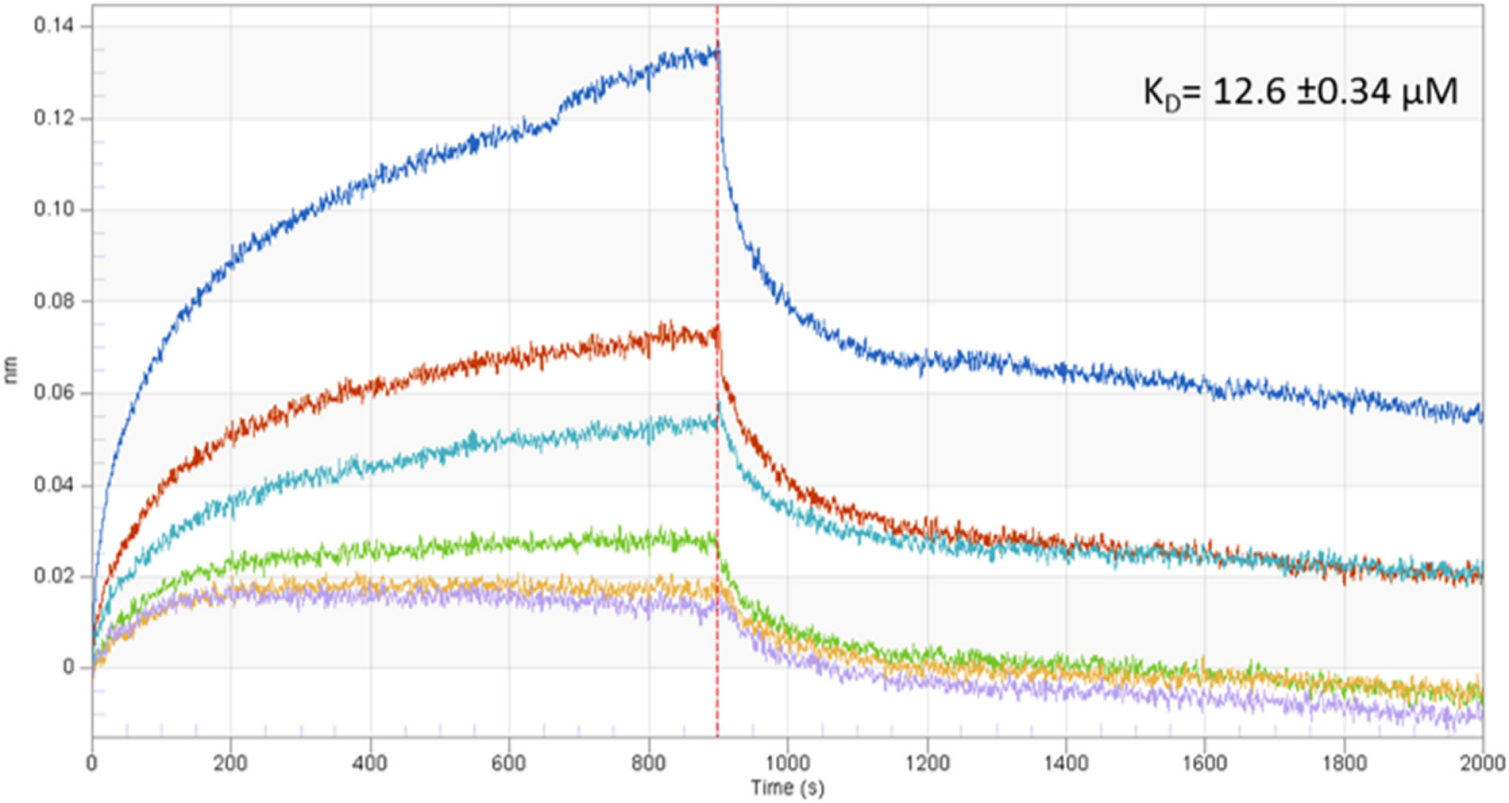
Binding affinity between APRc and human IgG. The binding affinity between APRc and human IgG was determined using biolayer interferometry (BLI). Human IgG was bound to anti-human IgG Fc-capture (AHC) biosensors and incubated with several dilutions, 0.42 μM (purple), 0.85 μM (yellow), 1.70 μM (green), 3.40 μM (light blue), 6.80 μM (orange), and 13.60 μM (dark blue), of APRc_110–231_His. The real-time binding response (nm) is plotted against time for different concentrations of APRc. Data were analyzed using the Data Analysis software (version 9.0; FortéBio) and a 1:1 binding interaction model with global fitting.

We next sought to determine the binding affinity between APRc and human IgG using biolayer interferometry (BLI). Supported by our results showing significantly higher APRc-human IgG interaction through the Fab region, we performed standard affinity measurements using anti-human IgG Fc-capture (AHC) biosensors. These sensors allowed for high-affinity immobilization of human IgG through the Fc domain, and we then measured the interaction with the recombinant soluble domain of APRc. Figure 6 shows real-time analysis results, demonstrating the specific interaction between APRc and human IgG with a binding affinity in the micromolar range (*K_D_* [binding affinity constant] of 12.6 ± 0.34 μM, coefficient of determination [*r*^2^] = 0.956), again confirming APRc as a novel nonimmune IgG-binding protein from rickettsiae.

### APRc domain binding with human IgG involves residues in the loop region 157 to 166.

**FIG 7:**
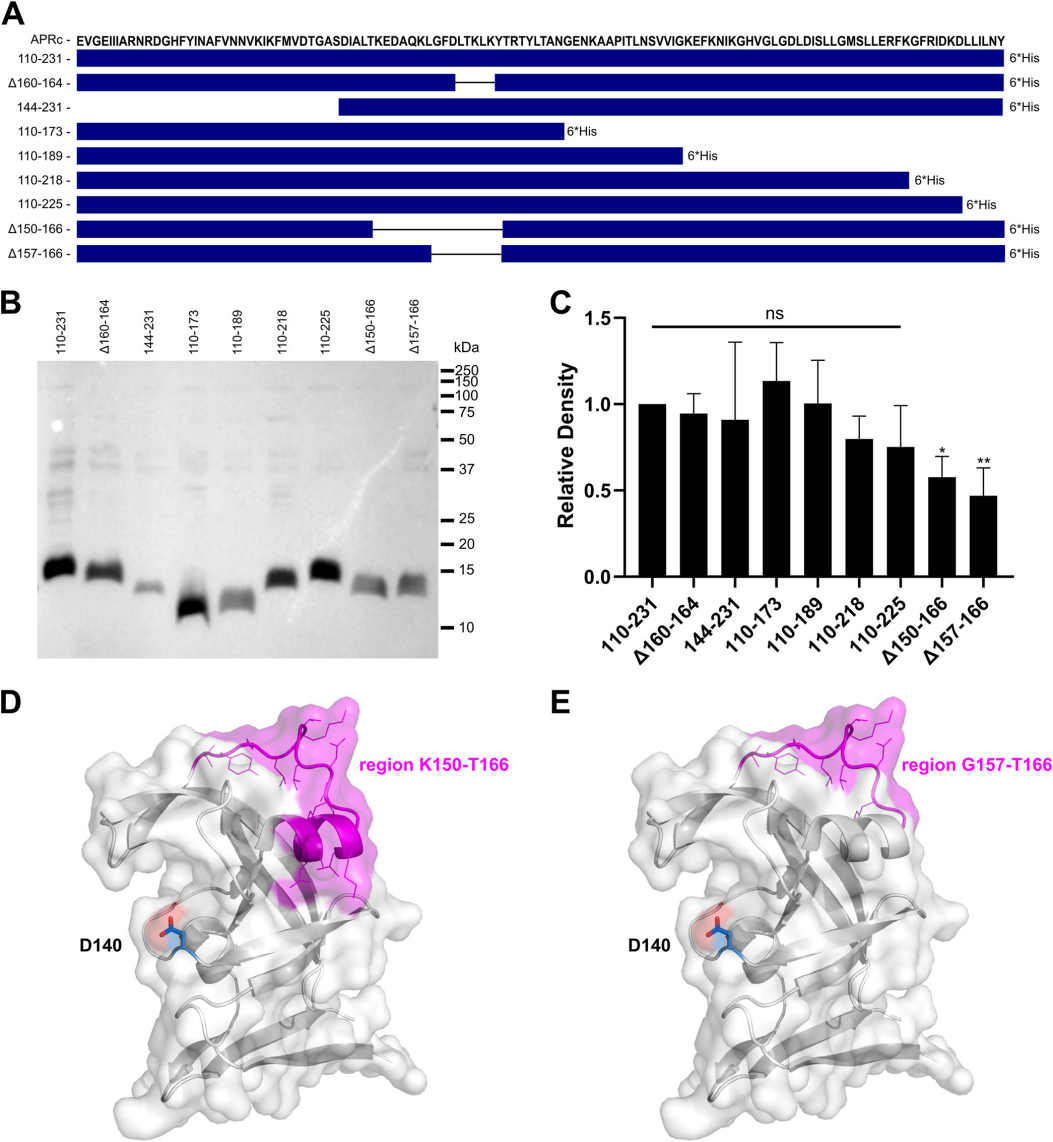
Binding to human IgG involves APRc domain residues in the loop region 157 to 166. (A) Amino acid sequence of the APRc soluble domain and schematic representation of the different truncated forms comprising deletions at each terminal as well as internal deletions, as follows: APRc_110–231_His, APRc(Δ160–164)_110–231_His, APRc_144–231_His, APRc_110–173_His, APRc_110–189_His, APRc_110–218_His, APRc_110–225_His, APRc(Δ150–166)_110–231_His, and APRc(Δ157–166)_100–231_His. (B and C) The soluble domain and the truncated forms of APRc were recombinantly expressed in E. coli. After expression, cells were harvested and normalized to an OD_600_ of 3, and equivalent amounts of total protein extracts were loaded onto SDS-PAGE gels and transferred to PVDF membranes. (B) Representative far-Western blot analysis with HRP-labeled rabbit IgG. (C) Densitometric analysis of IgG binding to APRc soluble domain and the respective truncated forms. The band intensity of each protein construct was normalized for the Coomassie blue staining of each corresponding lane. Data represent the mean ± standard deviation (SD) from 6 independent biological replicates, and the ratios were normalized for the APRc soluble domain construct, APRc_110–231_His. Significance was determined using a one-way ANOVA followed by Dunnett multiple-comparison test using GraphPad Prism 8 (ns, not significant; *, *P* < 0.05; **, *P* < 0.01). (D and E) Cartoon and surface representation of APRc (PDB ID, 5C9F) colored in white with alpha-helix and wide loop region 150 to 166 shown in magenta, with side chains of residues represented in line mode (D). (E) Only wide loop region 157 to 166 is highlighted in magenta for comparison. The catalytic aspartate (Asp140) is highlighted in blue and shown in stick mode. Evident surface exposure of the region 150 to 166 corroborates its major contribution to IgG complex formation. Images were generated using PyMOL (PyMOL molecular graphics system version 1.2r2; DeLano Scientific, LLC).

To identify the region(s) in APRc responsible for binding to IgG, we have generated several truncated forms of the soluble domain of the protease, comprising deletions at each terminal as well as internal deletions (Fig. 7A). The latter corresponds to various-size deletions of a wide loop, which assumes a very distinct conformation in APRc compared with all the other structures of retropepsins (37). The soluble domain and the truncated forms were expressed in E. coli, and the cells were harvested and normalized to an optical density at 600 nm (OD_600_) of 3 to load onto SDS-PAGE equivalent amounts of total protein extracts (Fig. S8A). We next evaluated binding to IgG by probing the transferred PVDF membrane with a rabbit IgG-HRP antibody. A negative control with E. coli BL21 Star(DE3) cells transformed with the empty vector (pET-23d) were used in parallel showing the specificity of nonimmune binding of rabbit IgG to APRc in these bacterial protein extracts (Fig. S8B). As shown in Fig. 7B and C, our results indicate that more than one region in APRc likely contributes to this interaction since binding was not abolished for any of the truncated forms here evaluated. However, a significant decrease in signal was observed for the recombinant forms with deletions in the region comprising amino acids 150 to 166, APRc(Δ150–166) and APRc(Δ157–166), anticipating this alpha-helix and wide loop region in APRc (Fig. 7D and E) as one of the regions contributing to complex formation. Interestingly, this loop is among the most variable domains in retropepsins and reported to grant diverse immunological properties to these proteases (40), corroborating its surface exposure.

10.1128/mBio.03059-21.8 FIG S8 Evaluation of the APRc region responsible for binding to immunoglobulins. (A) The soluble domain and the truncated forms of APRc [APRc_110–231_His, APRc(ΔLTKLK)_110–231_His, APRc_144–231_His, APRc_110–173_His, APRc_110–189_His, APRc_110–218_His, APRc_110–225_His, APRc(ΔGFDLTKLKYT)_100–231_His, APRc(ΔKEDAQKLGFDLTKLKYT)_110–231_His] were recombinantly expressed in E. coli. After expression, cells were harvested and normalized to an OD_600_ of 3, and equivalent amounts of total protein extracts (20 μl) were loaded onto SDS-PAGE gels followed by Coomassie blue staining. (B) E. coli BL21 Star(DE3) cells transformed with the empty vector pET-23d were treated under the same conditions as mentioned before and used in parallel as a negative control. Equivalent amounts of total protein extracts (20 μl) of E. coli expressing APRc_110–231_His and the empty vector (pET-23d) were then loaded onto SDS-PAGE gels followed by Coomassie blue staining (left panel) and evaluated by far-Western blotting with HRP-labeled rabbit IgG (right panel). Download FIG S8, TIF file, 1.9 MB. Copyright © 2021 Curto et al. 2021 Curto et al. https://creativecommons.org/licenses/by/4.0/ This content is distributed under the terms of the Creative Commons Attribution 4.0 International license.

### APRc binds IgG in human serum samples and targets additional serum components.

**FIG 8:**
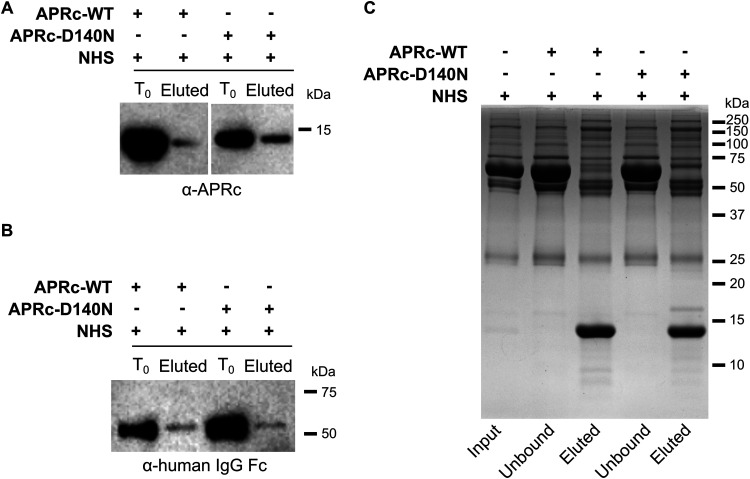
APRc binds IgG in human serum samples and targets additional serum components. The nonimmune APRc-IgG interaction in serum samples was further evaluated by immunoprecipitation (A) and pulldown (B) assays. (A) For the immunoprecipitation assay, the dimeric form of APRc (APRc_110–231_His) and the corresponding active site mutant [APRc_110–231_(D140N)His] were independently incubated with NHS (15× diluted in PBS), followed by incubation with protein A Mag Sepharose slurry. Magnetic beads were then washed, and protein elution was carried out by denaturation with SDS sample buffer diluted 6× in PBS. Detection of APRc in input (T_0_) and eluted samples (Eluted) was carried out by Western blotting with anti-APRc antibody. (B) For the pulldown analysis, the dimeric form of APRc (APRc_110–231_His) and the corresponding active site mutant [APRc_110–231_(D140N)His] were independently incubated with His Mag Sepharose Ni bead slurry. Upon binding, magnetic beads were incubated with NHS (15× diluted in PBS). After incubation, the beads were washed and protein was then eluted and denatured with SDS sample buffer diluted 6× in PBS. Detection of immunoglobulins in input (T_0_) and eluted samples (Eluted) was carried out by Western blotting with anti-human IgG Fc. (C) SDS-PAGE analysis followed by Coomassie blue staining of the samples from the pulldown assays: NHS sample without APRc (input), samples collected after sedimentation of the His Mag Sepharose Ni beads (unbound), and eluted samples (eluted).

Having confirmed the nonimmune APRc-IgG interaction using purified immunoglobulins, we evaluated if this interaction would also occur in the more complex context of normal human serum (NHS). For this purpose, we independently incubated the dimeric soluble domain of recombinant APRc wild type and active site mutant (both His tagged) with NHS diluted 15× in PBS and immunoprecipitated IgG with protein A Mag Sepharose beads. Upon washing, precipitated complexes were eluted in denaturing buffer and immunoblotted with an anti-APRc antibody. As shown in Fig. 8A, our results confirmed that both the wild-type and mutant forms of APRc were coimmunoprecipitated with IgG. Consistent with our observations with purified IgG, these results further corroborate that the nonimmune APRc-IgG binding is independent of the catalytic activity of the protease. As an additional confirmation for complex formation, we performed pulldown experiments using APRc wild-type and mutant forms as baits. Recombinant His-tagged APRc wild-type and mutant soluble domains were immobilized on His Mag Sepharose Ni beads, followed by incubation with NHS (diluted 15× in PBS). The bound proteins recovered from the nickel beads through elution with denaturing buffer were analyzed by Western blotting with an anti-human IgG Fc (HRP) antibody. The pulldown results are shown in Fig. 8B and confirm IgG as an APRc interacting partner. Similar to our immunoprecipitation (IP) results, both forms of APRc bound to human IgG.

The samples from the pulldown experiments were also analyzed with Coomassie blue-stained SDS-PAGE to assess for differences in bound proteins between incubations with the active and the inactive forms of APRc. Interestingly, our results show apparent differences in the profiles of the eluted proteins, namely, in the region between 50 kDa and 75 kDa, suggesting that APRc might bind and cleave additional serum components (Fig. 8C). Since several bacterial nonimmune IgG-binding proteins were also shown to interact with other serum proteins, likely to evade complement-mediated killing more effectively (16, 17), these results directed us to test whether APRc can mediate resistance to serum bactericidal activity.

### APRc protects E. coli from complement-mediated killing, and interaction with IgG contributes to serum resistance.

**FIG 9:**
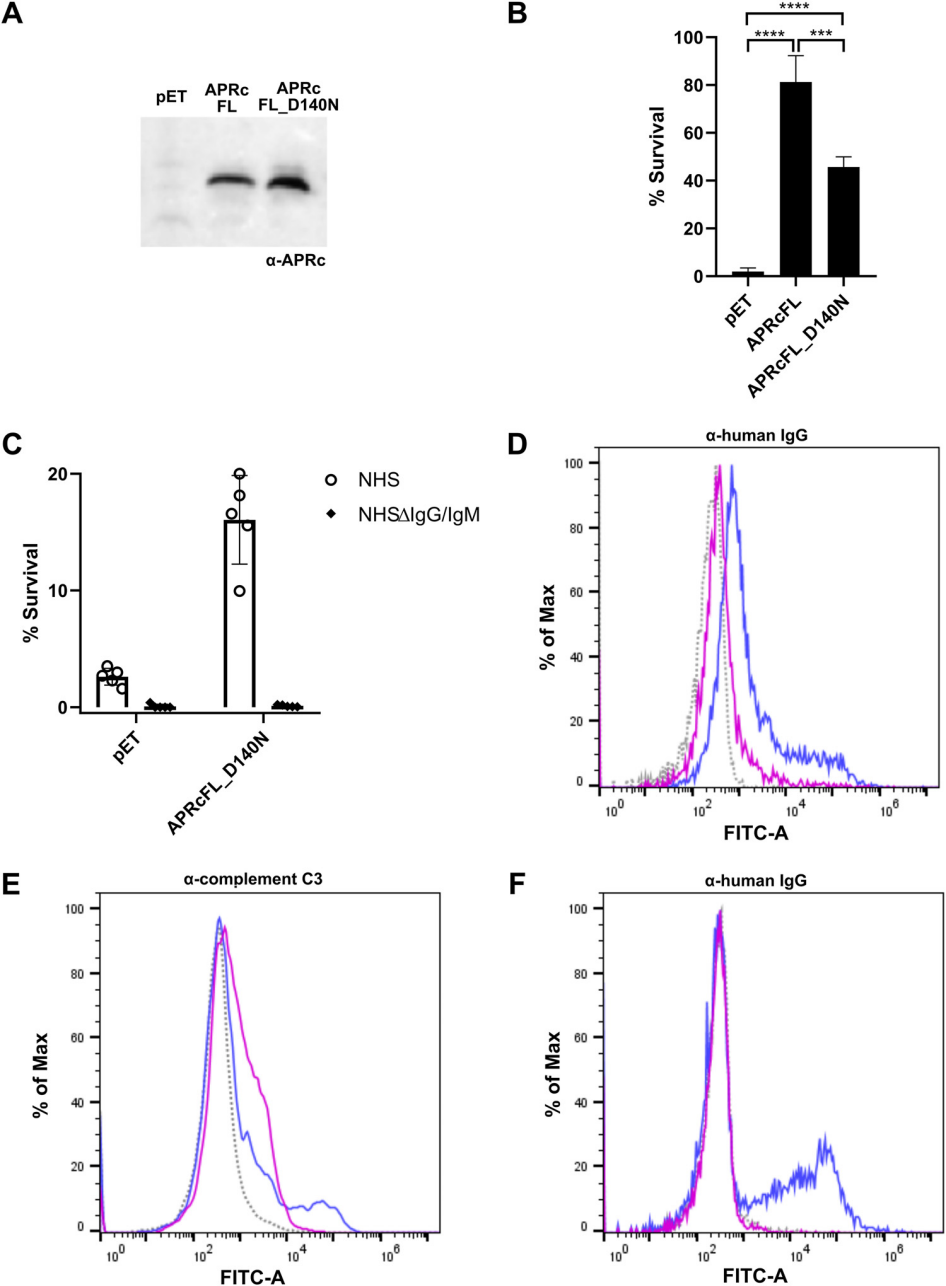
APRc protects E. coli from complement-mediated killing, and interaction with IgG contributes to serum resistance. (A) Western blot analysis with anti-APRc antibody of total protein extracts from BL21 Star(DE3) E. coli strain expressing the empty vector backbone pET28a (pET) or the plasmid encoding untagged full-length APRc wild type (APRcFL) and the corresponding catalytic mutant (APRcFL_D140N). (B) The serum-sensitive BL21 Star(DE3) E. coli strain expressing the empty vector (pET) or the plasmid encoding APRcFL and APRcFL_D140N was independently incubated 1:1 with PBS or in PBS containing 40% NHS for 1 h. After incubation, the samples were serially diluted, plated onto LB agar plates, and incubated overnight at 37°C. The average number of CFU per milliliter was calculated from the replicate plate counts. The data are presented as survival rate, which was calculated as the percentage of the original cell number at *T*_0_ (considered 100% survival). Data represent the mean ± SD from 4 independent biological replicates. Significance was determined using a one-way ANOVA followed by Tukey multiple-comparison test using GraphPad Prism 8 (***, *P* < 0.001; ****, *P* < 0.0001). (C) BL21 Star(DE3) E. coli strain expressing the empty vector (pET) or the plasmid encoding APRcFL_D140N was independently incubated 1:1 with PBS or in PBS containing 40% NHS or 40% NHSΔIgG/IgM for 1 h. After incubation, samples were treated as described for panel B. Data represent the mean ± SD from 4 independent biological replicates. (D) Flow cytometry analysis to query for human IgG deposition at the surface of E. coli cells. PFA-fixed E. coli expressing the untagged full-length APRc catalytic mutant (APRcFL_D140N) was incubated with HBSS (gray, dotted trace), HBSS containing 40% NHS (blue trace), and HBSS containing 40% NHSΔIgG/IgM (pink trace) for 1 h, followed by detection using anti-human IgG (Fc-specific)-FITC antibody. (E and F) Flow cytometry analysis querying for complement C3 (E) and IgG (F) deposition at the surface of *R. massiliae*. PFA-fixed *R. massiliae* was incubated with HBSS (gray, dotted trace), HBSS containing 50% NHS (blue trace), and HBSS containing 50% NHSΔIgG/IgM (pink trace) for 1 h, followed by detection using anti-complement C3 and secondary detection with goat anti-rabbit FITC-conjugated antibody or anti-human IgG (Fc-specific)-FITC antibody, respectively.

We have previously demonstrated the integration of full-length APRc into the outer membrane of E. coli, with the soluble catalytic domain extracellularly oriented (36). Therefore, to determine if APRc has a role in mediating serum resistance, we expressed the untagged full-length (FL) APRc wild type and the corresponding catalytic mutant (D140N) in the serum-sensitive BL21 Star(DE3) E. coli strain and assayed for survival upon incubation with serum. BL21 Star(DE3) cells transformed with the empty vector backbone (pET28a) were used as control. Four hours after protein induction, APRcFL wild-type-, APRcFL_D140N-overexpressing, and pET-control cells (Fig. 9A) were harvested and normalized to an OD_600_ of 0.2 in PBS and incubated 1:1 with PBS or in PBS containing 40% NHS for 1 h. The survival rate was calculated as the percentage of the cell number at time zero (considered 100% survival). Our results show that the expression of both forms of APRc resulted in significant differences in E. coli survival rates compared with cells transformed with the empty vector, with both forms of APRc promoting strong resistance to complement-mediated serum killing (Fig. 9B). Importantly, our results also indicate that the protective effect of APRc is significantly more pronounced in E. coli cells expressing the active form of the protease, anticipating that APRc proteolytically targets complement components critical for activation of the alternative pathway (as anticipated by our pulldown assays). Nevertheless, the protection observed for the catalytic dead mutant suggests that APRc-serum protein interactions not entailing cleavage still contribute significantly to serum resistance. To further address the relevance of Ig for this protective effect, APRcFL_D140N-overexpressing and pET-control bacteria were incubated with PBS or PBS containing 40% NHS, or 40% human IgG/IgM-depleted serum (NHSΔIgG/IgM). As shown in Fig. 9C, the protective effect of expressing the APRc mutant in E. coli is lost when the depleted serum is used, with cells becoming serum sensitive like those transformed with the empty vector. The differences in IgG deposition at the surface of APRcFL_D140N-overexpressing cells upon incubation with NHS or IgG/IgM-depleted serum were further confirmed by flow cytometry (Fig. 9D), and as expected, IgG deposition is increased in APRcFL_D140N-expressing cells compared with pET-control cells (Fig. S9). These results support the contribution of APRc-Ig interaction for the observed resistance mechanism. Taken together, these data show that APRc is sufficient to promote serum resistance, which, combined with the nonimmune APRc-IgG-binding activity, anticipates a multifunctional role for this rickettsial retropepsin in immune evasion. Supported by this evidence, we hypothesized that the deposition of the opsonin C3b at the surface of *Rickettsia* would differ upon incubation with NHS or NHSΔIgG/IgM. To investigate this, PFA-fixed *R. massiliae* incubated with Hanks’ balanced salt solution (HBSS) or HBSS containing 50% NHS or 50% NHSΔIgG/IgM was independently subjected to flow cytometry with an anti-C3 antibody or an anti-human Fc antibody, the latter as a control for IgG deposition. Indeed, in the absence of IgG/IgM, our results demonstrate an increased deposition of C3b at the surface of *Rickettsia* (Fig. 9E). Combined with the results shown in Fig. 9F, which provide additional confirmation for the association of human IgG at the surface of *Rickettsia*, these data strengthen our hypothesis that this interaction may provide a steric shield protecting *Rickettsia* from the deposition of complement and, thereby, contributing to immune evasion.

10.1128/mBio.03059-21.9 FIG S9 Flow cytometry analysis to query for human IgG deposition at the surface of E. coli BL21 Star(DE3) cells transformed with the empty vector pET28a. PFA-fixed E. coli bacteria transformed with pET28a were incubated with HBSS (gray, dotted trace), HBSS containing 40% NHS (blue trace), and HBSS containing 40% NHSΔIgG/IgM (pink trace) for 1 h, followed by detection using anti-human IgG (Fc-specific)-FITC antibody. Download FIG S9, TIF file, 0.4 MB. Copyright © 2021 Curto et al. 2021 Curto et al. https://creativecommons.org/licenses/by/4.0/ This content is distributed under the terms of the Creative Commons Attribution 4.0 International license.

## DISCUSSION

Many pathogenic bacteria use nonimmune Ig-binding proteins to help evade the host’s immune responses and establish infection. These proteins have been implicated in different functions related to pathogenic evasion mechanisms, such as inhibition of opsonophagocytic killing and complement inhibition (e.g., SpA, Sbi, protein H, group A streptococcus M proteins [16, 23, 41, 42]), B-cell superantigens (e.g., SpA, protein L [43, 44]), or B-cell activation (e.g., MID protein from Moraxella catarrhalis [19]). Therefore, Ig-binding proteins are considered important virulence factors in pathogenic bacteria and have been identified in Gram-positive (the majority) and Gram-negative bacteria (15, 17). With this work, we provide evidence that the retropepsin APRc is a novel Ig-binding protein and the first to be identified in *Rickettsia*. Moreover, we demonstrate that APRc is sufficient to promote serum resistance. Combined, these results anticipate APRc as a novel rickettsial evasin, adding to other immune-evasion tactics already known to be utilized by this obligate pathogen (10, 12, 13, 45, 46).

Our results confirm APRc binding to IgG from different species (rabbit IgG > human IgG > mouse IgG) as well as binding to different classes of human immunoglobulins (IgG > IgM > IgA). Furthermore, the affinity of APRc interaction to human IgG has been determined, revealing a *K_D_* (binding affinity constant) of 12.6 μM. This capacity to bind to different immunoglobulins is in line with what has been described for other nonimmune Ig-binding proteins, which generally display a great variety in reactivity. SpA and streptococcal protein G present different reactivities toward different subclasses of human and animal Ig. In addition to binding IgG, SpA also binds IgM and IgA (17), and protein L from *F. magna* shows reactivity toward all classes of immunoglobulins (25). In Gram-negative bacteria, the Ig-binding protein from H. pylori (Hsp60) shows reactivity toward human IgG and IgM but not toward IgA or rabbit and mouse IgG, whereas the Ig-binding protein from Y. pestis binds only human IgG but does not react with mouse, rabbit, and sheep IgG (17). Importantly, our ELISAs and far-Western analysis demonstrate that APRc binds preferentially to the F(ab′)_2_ region. Moreover, our results demonstrate that this interaction is independent of the catalytic activity of APRc and Ig binding does not entail cleavage under conditions close to optimal APRc cleavage conditions (36). Other proteins have been recognized as targeting the Fab fragment, such as SpA (targeting the VH3 subfamily) (47, 48), protein L (binding only to variable regions of k1, k3, and k4 light chains) (43, 49), the M. catarrhalis MID protein (targeting the CH1 region) (19), InvD from Yersinia pseudotuberculosis (selectively binding VH3/VK1) (27), and H. pylori Hsp60 (35). As described, all these proteins target different regions in the Fab domain, showing the existence of various modes of nonimmune interaction with this region of immunoglobulins. Therefore, we can only anticipate at this stage that APRc appears to bind preferably to conserved regions within mammalian Fab, requiring additional studies in the future to fully elucidate the details of such binding regions, including those on the Fc fragment, and its selectivity toward other Ig classes and subclasses.

When mapping the region of APRc involved in Ig binding, our results suggest that this interaction occurs through a very distinct wide loop (comprising residues 157 to 166) that presents a distinct conformation compared to all other known structures of retropepsins (37). However, since we could not identify a truncated form of APRc for which binding was completely abolished, this suggests that the interaction likely depends on more than one region in the protease. For most Ig-binding proteins for which the interaction domains have been identified, it has been reported that these domains comprise polypeptide repeats of several types that can vary in number and that the interaction can depend on more than one region for binding. This has been shown for SpA, streptococcus protein G, and protein L from *F. magna*, which was also shown to display two independent binding sites (with different affinities) to k light chains (17, 25, 50). Therefore, it is not totally unexpected that we could not fully map the interacting region in APRc. Interestingly, since APRc has no sequence or structural similarity with other reported Ig-binding proteins/domains, our results anticipate a new binding interface that requires additional structural characterization for deeper characterization. Moreover, the potential relevance of APRc as an affinity tool for protein purification warrants further investigation.

This nonimmune Ig-binding activity of APRc was also confirmed in human serum samples. As for the purified IgG, no apparent IgG cleavage activity was observed in these more complex samples. However, our results point toward cleavage of other serum components by APRc, and we have further demonstrated that expression of the full-length protease in E. coli is sufficient to promote resistance to complement-mediated killing. Interestingly, the expression of the catalytic mutant still significantly contributed to serum resistance, clearly suggesting that APRc likely interferes with the function of different serum components. In fact, many known Ig-binding proteins can also interact with other proteins of the host’s blood serum, sometimes to evade complement-mediated killing more effectively (16, 17, 51). For example, the IgA Fc-binding proteins from Streptococcus pyogenes, protein ARP and protein Sir (which also shows binding activity toward IgG), interact with the complement regulator C4BP, inhibiting complement deposition at the bacterial surface (52), and the staphylococcal IgG-binding protein Sbi also interacts with C3 and factor H, contributing to immune evasion through inhibition of opsonization and complement inhibition (23, 53). Recently, human IgG-protein H interaction was found to promote C4BP binding to this Ig-binding member of the M protein family, enhancing streptococcal immune evasion (54). Therefore, our evidence that APRc combines Ig-binding activity (independent of catalytic activity) with resistance to the bacteriolytic effects of complement anticipates a multifunctional role for this rickettsial protease. Our results showing that E. coli overexpressing the APRc mutant was rendered sensitive to serum killing upon incubation with IgG/IgM-depleted serum strengthen our hypothesis that nonimmune Ig-APRc binding may protect *Rickettsia* from the deposition of complement. Indeed, we confirmed an increase in C3b deposition at the surface of *Rickettsia* with antibody-depleted serum, further supporting the contribution of nonimmune IgG binding for serum resistance. Moreover, we cannot exclude that APRc-Ig binding may facilitate interaction with other complement regulators, or degradation of key complement components, thereby enhancing immune evasion. In fact, Riley and colleagues have recently demonstrated that Rickettsia australis is resistant to the lytic effects of complement and to complement-mediated opsonophagocytosis, anticipating the existence of rickettsial resistance mechanisms contributing to inhibit C3b deposition or to enhance C3 turnover (11). Curiously, they anticipated APRc as a candidate modulator of this process. Our observations on IgG deposition at the surface of *Rickettsia* and APRc-Ig interaction open the exciting hypothesis that APRc might indeed be involved in this resistance mechanism through nonimmune Ig binding (creating a protective shield) and in addition target complement components of the alternative pathway, also contributing to inhibition of membrane attack complex-mediated bacterial lysis. Although these hypotheses require further validation in the future, the results presented in this study strengthen the existence of additional complement evasion strategies in *Rickettsia*, apparently through different mechanisms. Moreover, since the Ig-binding proteins that target the Fab region (SpA, protein L, MDA) are known to act as B-cell or immunoglobulin superantigens (19, 43, 44), it is tempting to speculate that APRc might also target surface-exposed B-cell receptors through the exposed antibody segments. This potential interaction of APRc with components of the adaptive immune system and its functional consequences warrant further investigation.

In summary, APRc emerges as a novel moonlighting protein from *Rickettsia*, exhibiting different actions on serum components and likely acting as a novel evasin. Moonlighting proteins represent a vast group of virulence factors in many pathogenic bacteria (55). Interestingly, other rickettsial proteins have been shown to display moonlighting activity (e.g., OmpB [12, 45, 56, 57], OmpA [58, 59], Sca2 [60, 61], and PrsA and other secreted candidate moonlighting proteins [62, 63]), highlighting the importance of this strategy to expand *Rickettsia*’s virulence landscape. Our results further contribute to positioning this unique retropepsin as part of the arsenal of virulence factors utilized by *Rickettsia* to aid in host colonization. A following detailed analysis of APRc-Ig/complement interactions will illuminate how this strictly conserved protease contributes to pathogenesis and will be critical to dissect its significance as a future target for therapeutic intervention.

## MATERIALS AND METHODS

### Cell lines, *Rickettsia* growth, and purification.

Rickettsia montanensis isolate M/5-6T, *R. massiliae* isolate MTU5, *R. parkeri* isolate Portsmouth, and *R. africae* isolate ESF-5 T were obtained from CSUR-Collection de Souches de l’Unité des Rickettsies, Marseille, France, and were propagated in Vero cells and purified as previously described (9, 64). Vero cells were grown in Dulbecco’s modified Eagle’s medium (DMEM; Gibco) supplemented with 10% heat-inactivated fetal bovine serum (Gibco), 1× nonessential amino acids (Corning), and 0.5 mM sodium pyruvate (Corning). Uninfected and *Rickettsia*-infected Vero cells were maintained in a humidified 5% CO_2_ incubator at 34°C.

### Antibodies and protein conjugates.

Anti-APRc, rabbit polyclonal antibody raised toward the sequence Cys-Tyr-Thr-Arg-Thr-Tyr-Leu-Thr-Ala-Asn-Gly-Glu-Asn-Lys-Ala, was produced by GenScript. Anti-sAPRc, quail polyclonal antibody raised against the recombinant soluble domain of APRc (APRc110-231), was produced in-house at the CNC Avian Technological Unit. HRP-conjugated streptavidin (catalog no. 3999S) was purchased from Cell Signaling Technology. Mouse monoclonal anti-human Ig kappa light chain (MAB10050) was purchased from R&D Systems, Bio-techne. The rabbit polyclonal anti-mouse IgG (whole molecule)-peroxidase (A9044), rabbit polyclonal anti-chicken IgY (IgG) (whole molecule)-peroxidase (A9046), and anti-human IgG (Fc specific)-fluorescein isothiocyanate (FITC) antibody produced in goat (F9512) were purchased from Sigma-Aldrich. The complement C3 polyclonal antibody (PA5-21349) and the goat anti-rabbit IgG (H+L) cross-adsorbed secondary antibody, FITC (F-2765), from Invitrogen were purchased from Thermo Fisher Scientific. The mouse monoclonal anti-human IgG Fc(HRP) (A01854) and the mouse monoclonal anti-rabbit IgG (M205) (HRP) (A01827) were purchased from GenScript. Peroxidase AffiniPure mouse anti-goat IgG (H+L) (205-035-108) was purchased from Jackson ImmunoResearch. HRP labeling of the human IgG (I2511) and IgG fragments [human IgG Fab (009-0115) and F(ab′)_2_ (0009-0104) from Rockland; human IgG, Fc fragment (AG714) from Millipore] was carried out with the HRP conjugation kit (ab102890) from Abcam following the manufacturer’s instructions.

### Western blotting.

Samples were denatured with 6× SDS sample buffer (4× Tris-HCl, 30% glycerol, 10% SDS, 0.6 M dithiothreitol [DTT], 0.012% bromophenol blue, pH 6.8) during 10 min at 90°C, resolved by SDS-PAGE using 12.5% polyacrylamide gels in a Bio-Rad Mini-Protean Tetra cell, and transferred to a polyvinylidene difluoride (PVDF) membrane at 100 V during 100 min at 4°C. The membranes were blocked for 60 min with 2% BSA in Tris-buffered saline (TBS) containing 0.1% Tween 20 (TBS-T) and then incubated with the respective antibody. The following antibodies were used accordingly: mouse monoclonal anti-human IgG Fc antibody (HRP) (1:20,000 in TBS-T, 2% BSA); mouse monoclonal anti-rabbit IgG (M205) (HRP) (1:20,000 in TBS-T, 2% BSA); HRP-conjugated streptavidin (1:4,000 in TBS-T, 5% milk); rabbit polyclonal antibody anti-APRc (1:500 in TBS-T, 2% BSA); quail polyclonal antibody anti-APRc (1:2,000 in TBS-T, 2% BSA); mouse monoclonal anti-human Ig kappa light chain (1:259 in TBS-T, 2% BSA). The membranes were then washed again in TBS-T. Where applicable, membranes were incubated at room temperature for 1 h with the corresponding secondary antibodies: rabbit polyclonal anti-mouse IgG (whole molecule)-peroxidase (1:10,000 in TBS-T, 2% BSA); rabbit polyclonal anti-chicken IgY (IgG) (whole molecule)-peroxidase (1:2,000 in TBS-T, 2% BSA). After several washes in TBS-T, membranes were visualized by the enhanced chemiluminescence method using NZY Supreme ECL HRP substrate (NZYTech) on a VWR Imager (VWR).

### Nonimmune IgG-binding assays in SFG *Rickettsia*.

To detect nonimmune IgG-binding activity at the surface of intact bacteria, *R. africae* and *R. massiliae* (6.45 × 10^8^ *Rickettsia* bacteria) were fixed with 4% paraformaldehyde (PFA) for 20 min at room temperature. PFA-treated *Rickettsia* bacteria were then washed twice with 1 ml of phosphate-buffered saline (PBS) and incubated with 50 μl of human IgG (5.5 mg/ml), 50% normal human serum (NHS) (H6914; Sigma), or PBS as a control for 2 h at 37°C with rotation. After incubation, bacteria were washed twice with 1 ml of PBS. To elute proteins that bound at the surface of *Rickettsia*, bacteria were resuspended in 40 μl of PBS with 1 M NaCl and incubated for 20 min at room temperature with rotation. The suspension was then centrifuged at 13,000 × *g* for 3 min, and the supernatant was analyzed by Western blotting for the presence of human IgG.

To detect nonimmune IgG binding in total protein rickettsial extracts, purified *Rickettsia* samples were denatured using 6× SDS sample buffer during 10 min at 90°C. Total protein content in each sample was then quantified using the 2D Quant kit (GE Healthcare), and 12 μg of each *Rickettsia* species extract was analyzed by far-Western blotting for the presence of nonimmune IgG-binding proteins. For that, HRP-labeled human IgG (Sigma; I2511) was incubated in parallel with the purified recombinant monomeric form of APRc (APRc_110–231_-His) at a ratio of 1:550 mass/mass (m/m) (hIgG to APRc) for 3 h at room temperature with rotation or with the same volume of the respective buffer (20 mM HEPES, 100 mM NaCl, pH 7.4) as a control. Membranes were then probed with free and APRc-blocked HRP-labeled human IgG for 1 h at room temperature. After several washes in TBS-T, membranes were visualized in parallel by the enhanced chemiluminescence method using NZY Supreme ECL HRP substrate (NZYTech) on a VWR Imager (VWR). The integrated intensity of antibody-stained protein bands (≈24 kDa and 16 kDa) in rickettsial extracts incubated with free and APRc-blocked HRP-labeled human IgG was determined using the Image J software. The percentage of blocking was determined as follows: 100 − [(band intensity for APRc-blocked HRP-labeled human IgG/band intensity for HRP-labeled human IgG) × 100]. Experiments were performed in triplicate.

### Whole-cell ELISA.

To detect nonimmune IgG-binding activity at the surface of intact bacteria, *R. massiliae* was fixed with 4% PFA for 20 min at room temperature. PFA-treated *Rickettsia* bacteria were then washed 3 times with 1 ml of PBS and resuspended in PBS at 7.45 × 10^8^ bacteria per ml. PFA-fixed *R. massiliae* at 4.5 × 10^7^ bacteria per well or BSA at 1 μg/well was then applied as a coating onto Nunc MaxiSorp high-protein-binding-capacity 96-well ELISA plates (Thermo Fisher Scientific) overnight at room temperature. The wells were then washed 3 times with PBS containing 0.05% Tween (PBS-T) and blocked with PBS-T containing 3% BSA for 2 h at 37°C. The wells were washed 4 times with PBS-T and then incubated with different concentrations of HRP-labeled rabbit IgG (0 μg/ml, 10 μg/ml, and 50 μg/ml) diluted in PBS-T for 2 h at 37°C. The wells were then washed 4 times with PBS-T, and 100 μl of 1-Strep Ultra TMB ELISA substrate (Thermo Fisher Scientific) was added to each well and incubated at room temperature for 20 min, protected from light. To stop the reaction, 100 μl of a solution of 2 M sulfuric acid was added per well. The absorbance at 450 nm was measured in a BioTek PowerWave microplate spectrophotometer. Experiments were performed in triplicate, and significance was determined by an unpaired *t* test using GraphPad Prism v8.0.1 (GraphPad Software, Inc., CA, USA).

### DNA constructs.

All constructs used in this work are described in Table S1 in the supplemental material. The constructs pET_APRc_110–231_HisShort (pET-23d-based construct that comprises the coding sequence of APRc amino acids 110 to 231 fused with the C-terminal tag sequence HHHHHH); the active site mutant pET_APRc_110–231_(D140N)HisShort, where the active site aspartic acid residue was replaced by asparagine; and the construct pCoofy1_APRc_110–231_ (pCoofy-1-based construct [65] that includes the coding sequence of APRc amino acids 110 to 231 fused at the N terminus with a His tag followed by a human rhinovirus [HRV] 3C cleavage site) were already available in the laboratory.

10.1128/mBio.03059-21.10 TABLE S1 DNA constructs used in this study. Download Table S1, PDF file, 0.1 MB. Copyright © 2021 Curto et al. 2021 Curto et al. https://creativecommons.org/licenses/by/4.0/ This content is distributed under the terms of the Creative Commons Attribution 4.0 International license.

Different truncated forms of APRc were produced by PCR-based cloning using Phusion DNA polymerase (New England Biolabs [NEB]) and pET_APRc_110–231_HisShort as the template: construct pET_APRc_144–231_HisShort (construct encoding amino acids 144 to 231), forward primer 5′-GCCTCTGATATTGCACTGAC-3′ and reverse primer 5′-CATATGTATATCTCCTTCTTAAAGTTAAACAAA-3′; construct pET_APRc(Δ160–164)_110–231_HisShort (construct encoding amino acids 110 to 231 with the deletion of the sequence LTKLK, residues 160 to 164), forward primer 5′-TATACCCGTACGTACCTGACG-3′ and reverse primer 5′-ATCGAAACCCAGTTTCTGC-3′; construct pET_APRc(Δ157–166)_110–231_HisShort (construct encoding amino acids 110 to 231 with the deletion of the sequence GFDLTKLKYT, residues 157 to 166), forward primer 5′-CGTACGTACCTGACGGCC-3′ and reverse primer 5′-CAGTTTCTGCGCATCTTCTT-3′; construct pET_APRc(Δ150–166)_110–231_HisShort (construct encoding amino acids 110 to 231 with the deletion of the sequence KEDAQKLGFDLTKLKYT, residues 150 to 166), forward primer 5′-CGTACGTACCTGACGGCC-3′ and reverse primer 5′-CGTCAGTGCAATATCAGAGG-3′; construct pET_APRc_110–173_HisShort (construct encoding amino acids 110 to 173), forward primer 5′-CACCACCACCACCACCAC-3′ and reverse primer 5′-GTTGGCCGTCAG GTACGT-3′; construct pET_APRc_110–189_HisShort (construct encoding amino acids 110 to 189), forward primer 5′-CACCACCACCACCACCAC-3′ and reverse primer 5′-GCCGATAACCACGCTGTT-3′; construct pET_APRc_110–218_HisShort (construct encoding amino acids 110 to 218), forward primer 5′-CACCACCACCACCACCAC-3′ and reverse primer 5′-TTTAAAACGTTCCAGCAGAGAC-3′; construct pET_APRc_110–225_HisShort (construct encoding amino acids 110 to 225), forward primer 5′-CACCACCACCACCACCAC-3′ and the reverse primer 5′-ATCTTTATCGATGCGGAAACC-3′.

The construct used for the production of biotinylated APRc, comprising a 15-amino-acid avi tag peptide (biotin-accepting peptide) at the C terminus (pCoofy_HisAPRc_110–231_-avi), was assembled by sequence- and ligation-independent cloning (SLIC) using the pCoofy1_avi plasmid previously linearized by PCR using Phusion DNA polymerase (New England Biolabs) with the primer sets in parentheses (forward primer 5′-GGATCCGGACTGAACGACATCTTC-3′; reverse primer 5′-GGGCCCCTGGAACAGAACTTC-3′) and the PCR fragment obtained using the plasmid pET_APRc_110–231_His (37) as the template and the primer sets in parentheses (forward primer 5′-AAGTTCTGTTCCAGGGGCCCGAAGTTGGCGAAATTATCATT-3′ and reverse primer 5′-GAAGATGTCGTTCAGTCCGGATCCATAATTCAGAATCAGCAGATCTTTAT-3′). The recombination reaction was performed in a total volume of 10 μl using 100 ng of linearized vector in a vector/insert molar ratio of 1:3 using RecA (New England Biolabs). This vector encodes the soluble domain of APRc in frame with an N-terminal histidine tag followed by an HRV 3C protease cleavage site and fused in the C-terminal end with a biotin-accepting peptide (avi tag).

All amplification products were confirmed by agarose gel electrophoresis and digested with DpnI (NEB) for 2 h at 37°C, and the PCR products were liquid purified using the Macherey-Nagel NucleoSpin gel and PCR cleanup kit (Macherey-Nagel, Germany). Phosphorylation and ligation of DNA were performed for 3 h at room temperature in a single reaction, according to standard procedures (T4 DNA ligase and T4 polynucleotide kinase [PNK] were purchased from NEB). All positive clones were confirmed by sequencing.

### Expression and purification of the soluble forms of APRc.

The soluble domain of APRc fused to a C-terminal His tag (here named APRc_110–231_His) and the corresponding active site mutant protein were expressed by standard procedures (37). Briefly, E. coli BL21 Star(DE3) cells transformed with each plasmid construct, pET_APRc_110–231_HisShort and pET_APRc_110–231_(D140N)HisShort, were grown at 37°C until reaching an OD_600_ between 0.6 and 0.7 in the presence of ampicillin (100 μg/ml). Protein expression was then induced with 0.1 mM isopropyl-β-d-thiogalactopyranoside (IPTG) for 3 h at 30°C, after which cells were harvested by centrifugation at 9,000 × *g* for 20 min at 4°C, resuspended in 20 mM sodium phosphate (pH 7.4) buffer, containing 10 mM imidazole and 500 mM NaCl (buffer A), and frozen at −20°C. Bacteria were thawed at room temperature and lysed with a high-pressure homogenizer (Emulsiflex-C3; Avestin, ON, Canada). Bacterial lysates were then centrifuged at 24,000 × *g* for 20 min at 4°C followed by an ultracentrifugation at 315,594 × *g* for 20 min at 4°C. The resultant supernatant was then loaded onto a HisTrap HP 5-ml column (GE Healthcare Life Sciences) preequilibrated in buffer A. Protein elution was performed by a four-step gradient of imidazole (50 mM, 150 mM, 200 mM, and 500 mM) in 20 mM sodium phosphate (pH 7.4) buffer containing 500 mM NaCl. Fractions containing the protein of interest (200 mM imidazole gradient step) were pooled and buffer exchanged into 20 mM HEPES buffer (pH 7.4) by an overnight dialysis step. The dialyzed proteins were ultracentrifuged at 315,594 × *g* for 20 min at 4°C, and the supernatants were further purified in a Mono-S 5/50 GL column (Cytiva), previously equilibrated with 20 mM HEPES, pH 7.4. Protein elution was carried out with a linear gradient of NaCl (0 to 1 M) in 20 mM HEPES, pH 7.4. The oligomeric state of proteins was evaluated by analytical size exclusion chromatography in a Superdex 200 5/150 GL column (Cytiva), equilibrated in 20 mM HEPES (pH 7.4) containing 100 mM NaCl, using a Prominence Shimadzu high-pressure liquid chromatography (HPLC) system (Shimadzu, Tokyo, Japan) as described in reference 36. Proteolytic activity of purified forms of APRc was also confirmed by using the oxidized insulin β-chain as the substrate. Briefly, 2.5 μg of APRc_110–231_His and APRc_110–231_(D140N)His was incubated with 50 μg of oxidized insulin β-chain in 0.1 M sodium acetate, pH 6.0, overnight at 37°C, and digestion products were processed as described in reference 36.

For the production of the biotinylated form of APRc, E. coli BL21 Star(DE3) containing the plasmid pDW363ΔMBP, which enables the expression of biotin protein ligase (birA), was transformed with pCoofy1_HisAPRc_110–231_-avi and selected in LB medium with ampicillin (100 μg/ml) and kanamycin (50 μg/ml). Double-transformed E. coli cells were grown at 37°C until reaching an OD_600_ between 0.6 and 0.7 in the presence of ampicillin (100 μg/ml), kanamycin (50 μg/ml), and 50 μM biotin. Protein expression was then induced overnight with 0.1 mM IPTG at 20°C, after which cells were harvested by centrifugation at 9,000 × *g* for 20 min at 4°C, resuspended in buffer A, and frozen at −20°C. Bacteria were thawed and processed as described above for loading onto a HisTrap HP 5-ml column. Protein elution was performed by the four-step gradient of imidazole as previously described. The fractions containing the protein of interest (150 mM imidazole step) were pooled and buffer exchanged into 20 mM Tris-HCl (pH 8.0) by an overnight dialysis step. The dialyzed proteins were ultracentrifuged at 315,594 × *g* for 20 min at 4°C, and the resultant supernatants were further purified in a Mono-Q 5/50 GL column (Cytiva), equilibrated with 20 mM Tris-HCI buffer, pH 8.0. Protein elution was carried out with a linear gradient of NaCl (0 to 1 M) in 20 mM Tris-HCI, pH 8.0. To remove the N terminus hexahistidine tag, the eluted protein fractions were incubated overnight with 2 μl HRV 3C protease in 50 mM Tris-HCl (pH 7.5) containing 150 mM NaCl, at 4°C. After digestion, samples were then incubated with Ni Sepharose high-performance beads (GE Healthcare) for 15 min at room temperature with agitation. After incubation, protein samples were filtered through 0.2-μm filters and dialyzed against PBS for 3 h at 4°C.

For the production of the untagged soluble domain of APRc (APRc_110–231_), E. coli BL21 Star(DE3) cells transformed with pCoofy1_APRc_110–231_ were grown at 37°C until reaching an OD_600_ between 0.6 and 0.7 in the presence of kanamycin (50 μg/ml). Protein expression was then induced overnight with 0.1 mM IPTG at 20°C, after which cells were harvested by centrifugation at 9,000 × *g* for 20 min at 4°C, resuspended in buffer A, and frozen at −20°C. Bacteria were processed as described above, and the resultant supernatant was loaded onto a HisTrap HP 5-ml column. Protein elution was performed by the four-step gradient of imidazole. Fractions containing the protein of interest (150 mM and 500 mM imidazole step) were pooled and buffer exchanged into PBS, pH 7.4. The dialyzed proteins were ultracentrifuged at 315,594 × *g* for 20 min at 4°C, and the resultant supernatants were further applied to a HiLoad 26/60 Superdex 200 gel filtration column (Cytiva), previously equilibrated in PBS, pH 7.4. The protein fractions were pooled and further concentrated using an Amicon Ultra-15 centrifugal filter unit (Millipore). To remove the N terminus hexahistidine tag, the eluted protein was incubated overnight with HRV 3C protease in an m/m ratio of 1:33 (3C to APRc) in 50 mM Tris-HCl (pH 7.5) containing 150 mM NaCl at 4°C. After digestion, samples were then incubated with Ni Sepharose high-performance beads (GE Healthcare) for 15 min at room temperature with agitation. After incubation, samples were filtered through an 0.45-μm Spin-X centrifuge tube filter (Corning, Costar, Spin-X) and dialyzed against PBS for 3 h at 4°C.

All chromatographic steps were carried out in a BioLogic DuoFlow system. Protein concentration was estimated using a NanoDrop 1000 spectrophotometer (Thermo Fisher Scientific).

### Far-Western blotting.

The ability of APRc to bind IgGs from different origins and to bind to F(ab′)_2_, F(ab), and Fc fragments was evaluated by far-Western blotting. For that, different amounts (0.5, 2, 5, and 10 μg) of recombinant purified untagged APRc (APRc_110–231_) were resolved by SDS-PAGE using 12.5% polyacrylamide gels and transferred to a PVDF membrane at 100 V during 100 min at 4°C. The membranes were blocked for 60 min with 2% BSA in TBS-T and then incubated with the respective antibody: 4.5 μg of HRP-labeled human IgG (I2511; Sigma-Aldrich), 4.5 μg of HRP-labeled rabbit IgG (A9044; Sigma-Aldrich), and 1.2 μg of mouse IgG (205-035-108; Jackson ImmunoResearch). For the evaluation of binding to IgG fragments, membranes were incubated with 4.5 μg of HRP-labeled rabbit F(ab′)_2_, HRP-labeled human F(ab′)_2_, HRP-labeled human F(ab′), and HRP-labeled human Fc. Membranes were washed in TBS-T and visualized by the enhanced chemifluorescence (ECF) method using ECF substrate (GE Healthcare) on a Molecular Imager FX system (Bio-Rad) or using NZY Supreme ECL HRP substrate (NZYTech) on a VWR Imager (VWR), depending on the antibody used.

### ELISA.

The interaction between APRc and immunoglobulins from different origins and classes was evaluated by sandwich enzyme-linked immunosorbent assay (ELISA). Nunc MaxiSorp high-protein-binding-capacity 96-well ELISA plates (Thermo Fisher Scientific) were coated with 1 μg/well of BSA (negative control) and 1 μg/well of each immunoglobulin (human IgG [I2511; Sigma-Aldrich], mouse IgG [I5381; Sigma-Aldrich], rabbit IgG [produced by GenScript {66}], human IgA [I4036; Sigma-Aldrich], human IgM [I8260; Sigma-Aldrich], and the mouse monoclonal antibody [A00186; GenScript]). To evaluate the interaction between APRc and the different human IgG fragments, the plates were coated with 1 μg/well of BSA (negative control) and 1 μg/well of APRc_110–231_His. The plates were left for 2 h at 37°C. All wells were then washed 3 times with PBS containing 0.05% Tween 20 (PBS-T) and blocked with PBS-T buffer containing 3% BSA for 2 h at 37°C. The wells were washed 4 times with PBS-T. The plates coated with Ig were incubated with different amounts of biotinylated APRc (0 μg/well, 1 μg/well, 2.5 μg/well, 10 μg/well, 22.5 μg/well, 30 μg/well, and 40 μg/well) diluted in PBS for 1 h at 37°C. The plates coated with APRc were incubated with 9.09 pmol/well of HRP-labeled human F(ab′)_2_, F(ab′), and Fc. The wells were washed 4 times with PBS-T. For APRc-Ig interaction, the plates were further incubated with HRP-conjugated streptavidin (Cell Signaling Technology) (1:2,000) in PBS-T buffer containing 3% milk for 1 h at 37°C. The wells were then washed 4 times with PBS-T, and 100 μl of 1-Strep Ultra TMB ELISA substrate (Thermo Fisher Scientific) was added to each well and incubated at room temperature for 20 min, protected from light. To stop the reaction, 100 μl of a solution of 2 M sulfuric acid was added per well. The absorbance at 450 nm was measured in a BioTek PowerWave microplate spectrophotometer. Experiments were performed in triplicate, and significance was determined by two-way analysis of variance (ANOVA) followed by Tukey multiple-comparison test using GraphPad Prism v8.0.1 (GraphPad Software, Inc., CA, USA).

### Affinity determination by biolayer interferometry.

The interaction between APRc and IgG was performed on an Octet Red96 biolayer interferometry system (FortéBio Inc., Menlo Park, CA, USA) using anti-human IgG Fc capture (AHC) biosensors. The assays were performed at 30°C, in a total reaction volume of 200 μl and using 96-well plates (Greiner Bio, Germany). First, human IgG (I8640; Sigma-Aldrich) was loaded onto the AHC biosensors at 15 μg/ml in 1× assay buffer (20 mM HEPES, pH 7.4, 170 mM NaCl, 0.02% Tween 20, 0.1% BSA) and then incubated with different concentrations of APRc_110–231_His. The analyte was diluted also in the same buffer and tested in a range from 0.42 μM to 13.6 μM prepared by half serial dilutions. The kinetic parameters and affinities were calculated using a buffer blank as reference with a nonlinear global fit of the data, using Octet data analysis software version 9.0 (FortéBio Inc., Menlo Park, CA, USA).

### Production of different IgG fragments.

Human and rabbit F(ab′)_2_ fragments were produced by incubation of human IgG (I2511) at 0.79 mg/ml and rabbit IgG (A9044) at 0.79 mg/ml, with 0.04 mg/ml of pepsin (P6887; Sigma-Aldrich) in 50 mM sodium acetate (pH 4.0) containing 100 mM NaCl, at 37°C for 8 h 30 min. Reaction was then stopped by the addition of 10 μM pepstatin A. Sample was then diluted with 1 ml of PBS and concentrated in a Vivaspin 500 (50-kDa polyethersulfone [PES] column; Sartorius) previously equilibrated with PBS. To remove the remaining Fc fragments, samples were incubated for 2 h with protein A Mag Sepharose bead slurry (Cytiva) at room temperature with agitation. After incubation, the beads were separated with a MagRack 6, and the supernatant containing the F(ab′)_2_ was collected and analyzed by SDS-PAGE and Western blotting with anti-human Ig kappa light chain and anti-human Fc antibodies.

Human F(ab′) fragments were produced by incubation of human F(ab′)_2_ at 1.26 mg/ml with papain (P4762; Sigma-Aldrich) at 0.2 mg/ml in PBS (pH 7.4) containing 0.02 M EDTA and 0.02 M cysteine at 37°C for 1 h 30 min. The sample was then diluted in 20 ml of PBS, pH 7.4, and concentrated in an Amicon Ultra-4 centrifugal filter unit (Ultracel 3,000 Da) (Merck), previously equilibrated in PBS, pH 7.4. To remove the Fc fragments, the sample was incubated for 2 h with protein A Mag Sepharose bead slurry (GE Healthcare) at room temperature with agitation. After incubation, the beads were separated with a MagRack 6, and the supernatant containing the F(ab′) fragments was collected, treated with 10 μM E-64 protease inhibitor, and analyzed by SDS-PAGE and Western blotting with anti-human Ig kappa light chain and anti-human Fc antibodies for quality control.

### Cross-linking reactions with glutaraldehyde.

To evaluate APRc-IgG interactions, cross-linking reactions were performed. For that, recombinant biotinylated APRc_110–231_, APRc_110–231_His, and its corresponding active-site mutant APRc_110–231_(D140N)His were incubated with human IgG or human F(ab′)_2_ fragments at an equimolar ratio (166.85 pmol) for 4 h at 37°C. After incubation, glutaraldehyde was added to a final percentage of 0.1% (vol/vol) and incubated for 4 min at 37°C. As a control, similar incubations were treated with 2 μl of water. The reactions were stopped by the addition of 2 μl of the quenching buffer, 1 M Tris-HCl, pH 8.0. The samples were then denatured with 6× SDS sample buffer for 10 min at 90°C and analyzed by Western blotting with HRP-streptavidin or with rabbit anti-APRc antibody.

### Nonimmune IgG-binding and cleavage assays.

To evaluate if APRc combines IgG binding with IgG-cleavage activity, incubations of recombinant APRc with human IgG were carried out. The dimeric form of APRc (APRc_110–231_His) and its corresponding active site mutant were incubated with human IgG (I2511) at an equimolar ratio (333.7 pmol) in PBS (pH 7.4) for 4 h at 37°C. After incubation, samples were denatured under nonreducing conditions with 6× loading buffer (4× Tris-HCl, 30% glycerol, 10% SDS, 0.012% bromophenol blue, pH 6.8) during 10 min at 90°C and resolved by SDS-PAGE using 10% polyacrylamide gels for Western blotting with anti-APRc antibody and mouse monoclonal anti-human IgG Fc (HRP) antibody.

### Nonimmune APRc-human IgG binding in the context of NHS.

The nonimmune APRc-Ig interaction in serum samples was evaluated by pulldown and immunoprecipitation assays. For the pulldown assays, 85 μg of the dimeric form of APRc (APRc_110–231_His) and of the corresponding active site mutant [APRc_110–231_(D140N)His] was diluted in PBS and independently incubated with 25 μl of His Mag Sepharose Ni bead slurry (Cytiva) for 1 h 30 min, at room temperature, with agitation. Upon binding, the magnetic beads loaded with APRc were separated with a MagRack 6, washed three times with 1 ml of PBS buffer, and then incubated with NHS (Sigma-Aldrich) diluted 15× in PBS, for 4 h at 37°C with agitation. After incubation, the beads were washed three times with 1 ml of PBS buffer, 1 ml of PBS buffer containing 200 mM NaCl, and 1 ml of PBS buffer containing 0.1% Tween. Lastly, 50 μl of elution buffer (SDS sample buffer diluted 6× in PBS) was added to the magnetic beads and denatured for 10 min at 90°C. The beads were separated with a MagRack 6, the supernatant was collected, and the proteins were resolved by SDS-PAGE for Coomassie blue staining and Western blot analysis with mouse anti-human IgG Fc (HRP) antibody. For the immunoprecipitation assay, NHS (Sigma) diluted 15× in PBS was incubated with 62.5 μg of the dimeric form of APRc (APRc_110–231_His) or of the corresponding active site mutant [APRc_110–231_(D140N)His], for 4 h at 37°C. Subsequently, these suspensions were incubated for 35 min, with agitation, at room temperature, with 40 μl of protein A Mag Sepharose slurry (Cytiva). After incubation, the resin was separated with a MagRack 6 and washed three times, with 1 ml of TBS buffer, 1 ml of TBS buffer containing 250 mM NaCl, and 1 ml of TBS buffer containing 1% octyl-β-glucoside. Lastly, 50 μl of elution buffer was added to the magnetic beads and denatured for 10 min at 90°C. The beads were separated with a MagRack 6, the supernatant was collected, and the proteins were resolved by SDS-PAGE for Coomassie blue staining and Western blot analysis with rabbit anti-APRc antibody.

### Small-scale expression screening and mapping of Ig-interacting domain.

For small-scale expression screening and mapping of Ig-interacting domain, the following truncated forms of APRc were used: APRc_110–231_His, APRc_144–231_His, APRc(ΔLTKLK)_110–231_His, APRc(ΔGFDLTKLKYT)_110–231_His, APRc(ΔKEDAQKLGFDLTKLKYT)_110–231_His, APRc_110–173_His, APRc_110–189_His, APRc_110–218_His, and APRc_110–225_His. E. coli BL21 Star(DE3) cells transformed with each plasmid construct were grown in 1 ml of LB containing 100 μg/ml ampicillin overnight at 37°C with agitation. E. coli BL21 Star(DE3) cells transformed with the empty vector pET-23d were used in parallel as a negative control. The preinocula were then diluted in 2 ml of LB containing 100 μg/ml ampicillin to an initial OD_600_ of 0.05 and then grown at 37°C with agitation in 24-deep-well plates, until reaching an OD_600_ of 0.6. Protein expression was then induced overnight with 0.1 mM IPTG at 20°C. Before cell lysis, E. coli density was assessed. For protein extraction, a cell density corresponding to an OD_600_ of 3 (per condition) was harvested by centrifugation at 16,000 × *g* for 5 min, at room temperature. The pellets were resuspended in BugBuster protein extraction reagent (Novagen) for 20 min at room temperature with agitation. The samples were then denatured with 6× SDS sample buffer for 10 min at 90°C and resolved by SDS-PAGE for Coomassie blue staining and analysis by far-Western blotting with rabbit anti-mouse IgG (HRP) antibody. Densitometric analysis of IgG binding to APRc soluble domain and the respective truncated forms was performed using the Image J software. The band intensity of each protein construct was normalized for the Coomassie blue staining of each corresponding lane, and the ratios were normalized for the APRc soluble domain construct, APRc_110–231_His. Experiments were performed as 6 independent biological replicates.

### Serum resistance.

For serum sensitivity assays, E. coli BL21 Star(DE3) cells transformed with the plasmid constructs pET28a_APRcFL and pET28a_APRcFL_D140N (pET28a-based vector encoding the full-length sequence of APRc [amino acids 1 to 231] and its corresponding active site mutant, respectively) (36) and the control plasmid pET28a were grown at 37°C, in the presence of kanamycin (50 μg/ml), until reaching an OD_600_ between 0.6 and 0.7. Protein expression was then induced with 0.1 mM IPTG, for 3 h at 30°C. Upon expression, bacteria were resuspended in PBS to an OD_600_ of 0.2, and 100 μl of this suspension was diluted 1:1 with PBS, 40% NHS, 40% human IgG/IgM-depleted serum (NHSΔIgG/IgM) (34010-10; Pel-Freez Biologicals LLC), followed by incubation for 1 h at 37°C with rotation. After incubation, the samples were serially diluted, plated onto LB agar plates, and incubated overnight at 37°C. The average number of CFU per milliliter was calculated from the replicate plate counts. The data are presented as survival rate, which was calculated as the percentage of the original cell number at *T*_0_ (considered 100% survival). Independent quadruplicate samples were processed per experimental condition. Confirmation of protein expression was performed for all replicates. To this end, a cell density corresponding to an OD_600_ of 1 (per experimental condition) was harvested by centrifugation at 16,000 × *g* for 5 min, at room temperature. The pellets were resuspended in BugBuster protein extraction reagent (Novagen) for 20 min at room temperature with agitation. The samples were then denatured with 6× SDS sample buffer for 10 min at 90°C and resolved by SDS-PAGE for Western blot analysis with rabbit anti-APRc antibody.

### Flow cytometry.

To detect C3 and IgG at the surface of *Rickettsia*, *R. massiliae* bacteria were fixed with 4% PFA for 20 min at room temperature. PFA-treated *Rickettsia* bacteria were washed twice with 1 ml of PBS and resuspended in 100 μl of Hanks’ balanced salt solution (HBSS), 50% NHS, or 50% NHSΔIgG/IgM in HBSS, followed by incubation for 1 h at 37°C with rotation. After incubation, the samples were washed twice with 1 ml of cold PBS. For detection of IgG at the surface of E. coli, bacteria transformed with the plasmid construct pET28a_APRcFL_D140N and the control plasmid pET28a were subjected to protein expression as described above (“Serum resistance”). Upon expression, an equivalent of an OD_600_ of 1 per ml (per condition) was fixed with 4% PFA for 20 min at room temperature and washed twice with 1 ml of cold PBS. Bacteria were directly resuspended in 100 μl of HBSS, 50% NHS, or 50% NHSΔIgG/IgM in HBSS, followed by incubation for 1 h at 37°C with rotation. After incubation, the samples were washed twice with 1 ml of PBS. All samples were blocked with PBS containing 2% BSA for 2 h at 37°C. Samples were incubated with anti-complement C3 (1:100 in PBS, 2% BSA) or with anti-human IgG (Fc specific)-FITC (1:500) antibodies for 1 h at room temperature, followed by washing with PBS (3×). Samples probed with anti-C3 were subsequently labeled with goat anti-rabbit FITC-conjugated antibody, followed by washing with PBS (3×). Bacteria were analyzed by flow cytometry using a Becton Dickinson Accuri C6 cell analyzer instrument and FlowJo software.
