# Supplementary figures and images for: The Retropepsin-Type Protease APRc as a Novel Ig-Binding Protein and Moonlighting Immune Evasion Factor of Rickettsia
Source: mBio. 2021 Dec 7;12(6):e03059-21. doi: 10.1128/mBio.03059-21 (PMC8649778; doi:10.1128/mBio.03059-21)

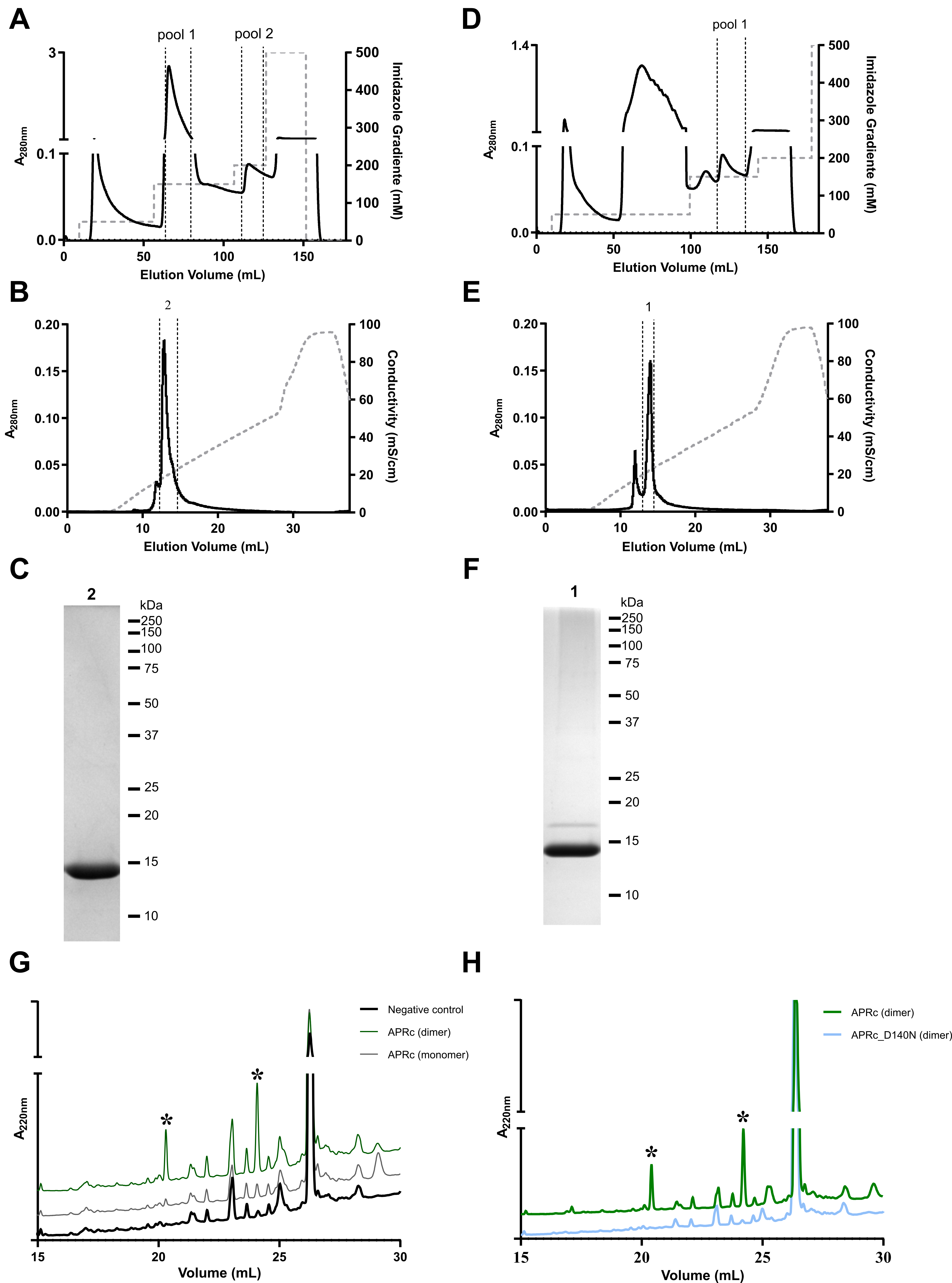

Supplement: FIG S1 [file mbio.03059-21-sf001.tif]

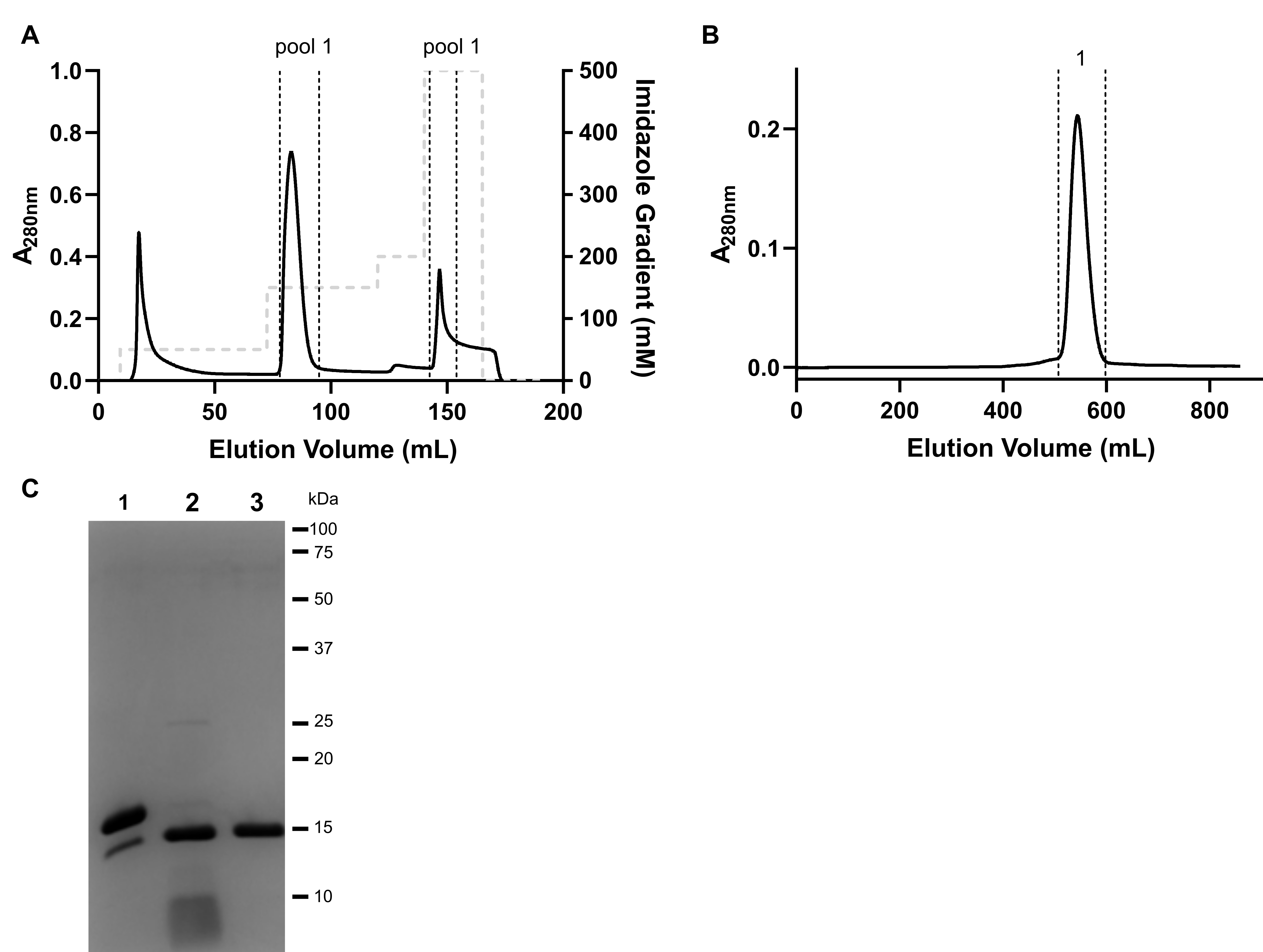

Supplement: FIG S2 [file mbio.03059-21-sf002.tif]

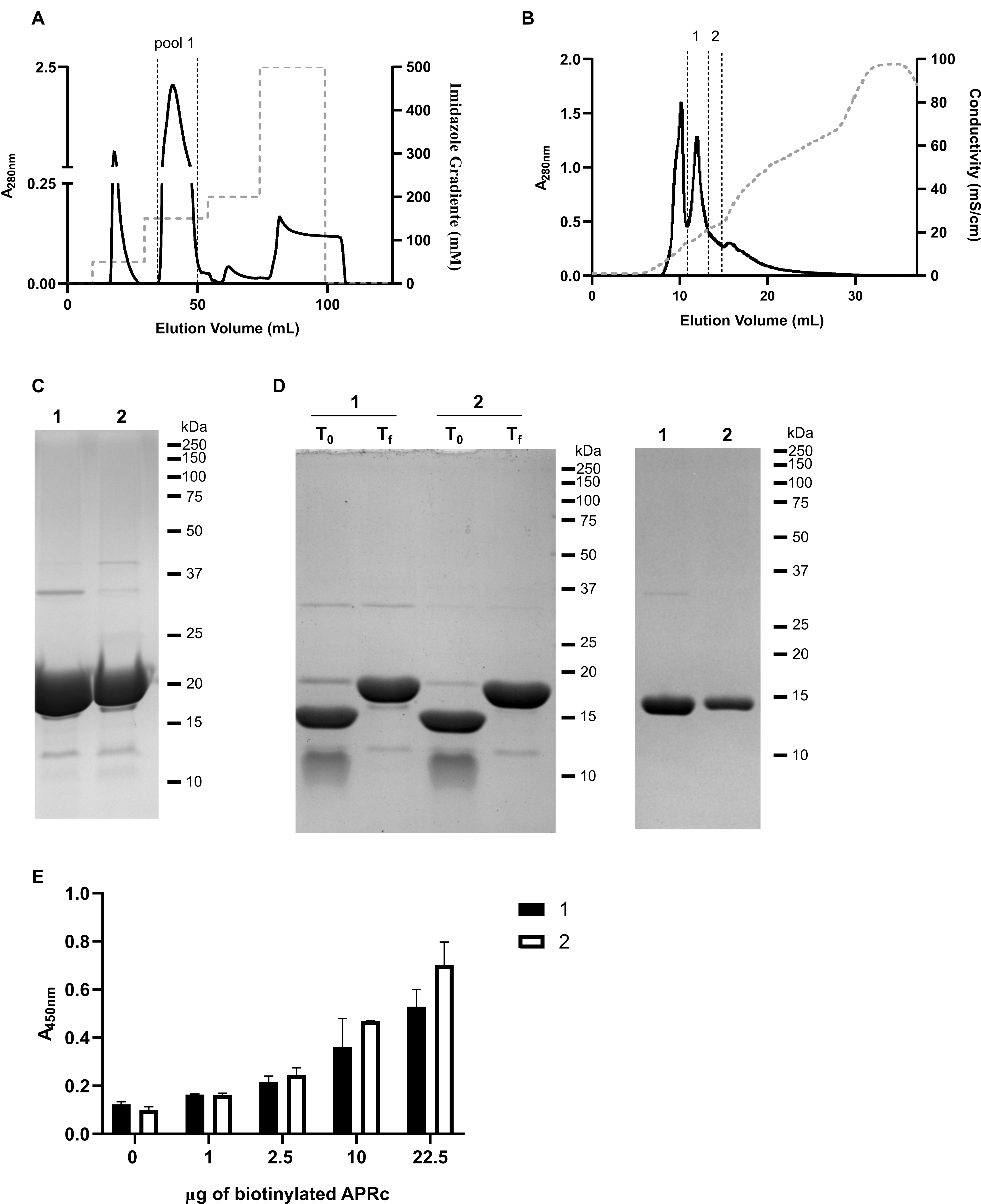

Supplement: FIG S3 [file mbio.03059-21-sf003.tif]

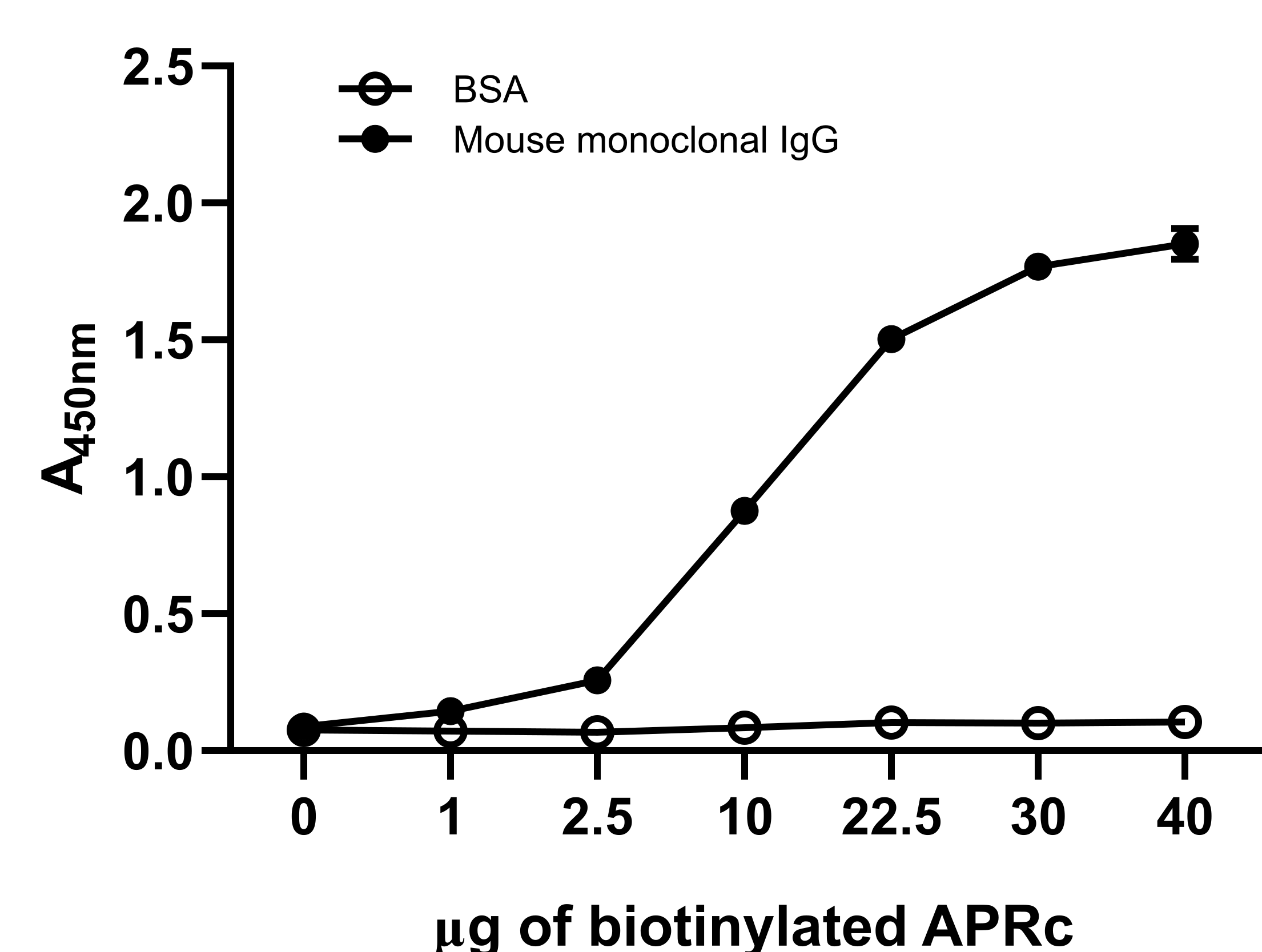

Supplement: FIG S4 [file mbio.03059-21-sf004.tif]

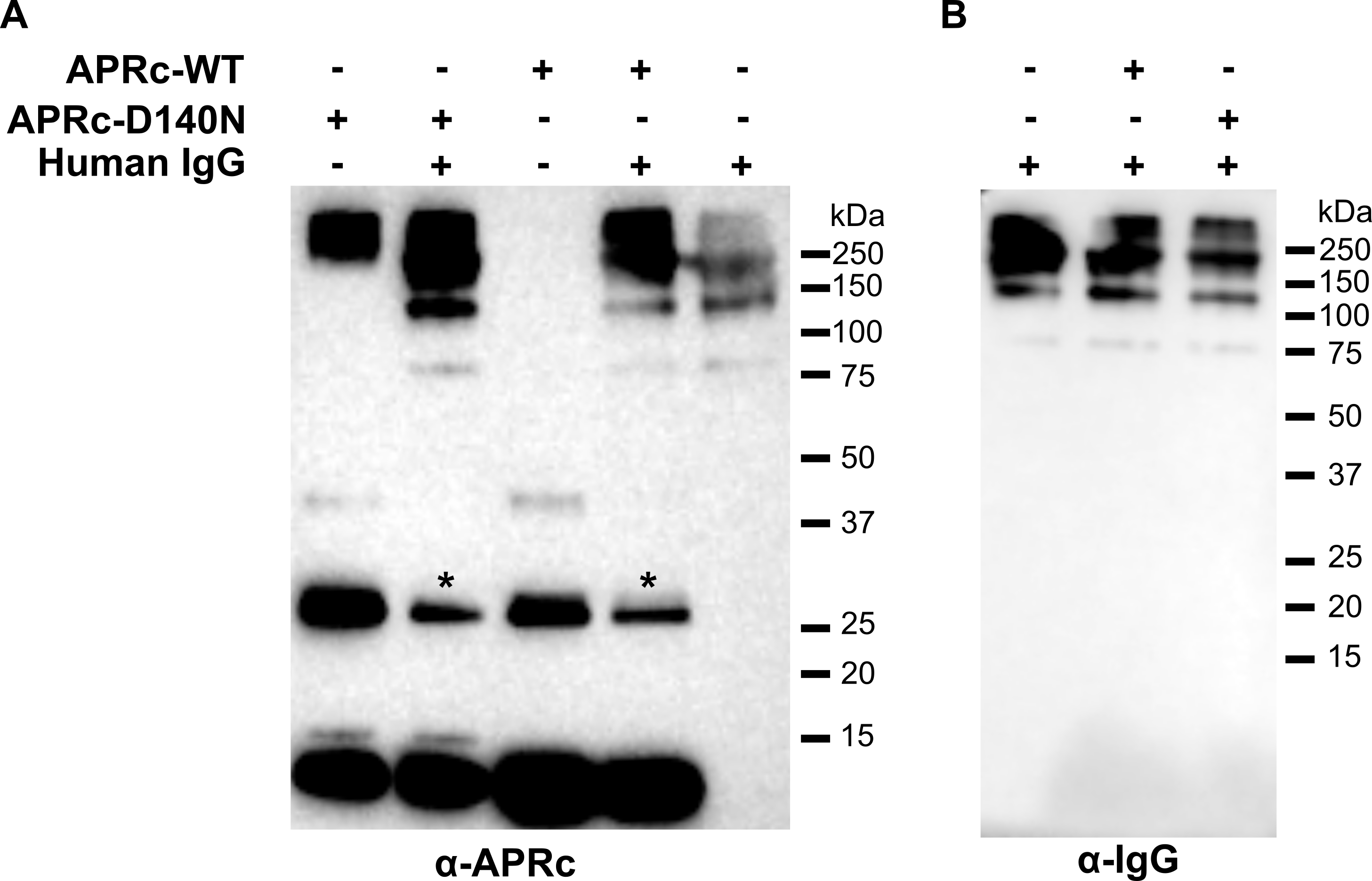

Supplement: FIG S5 [file mbio.03059-21-sf005.tif]

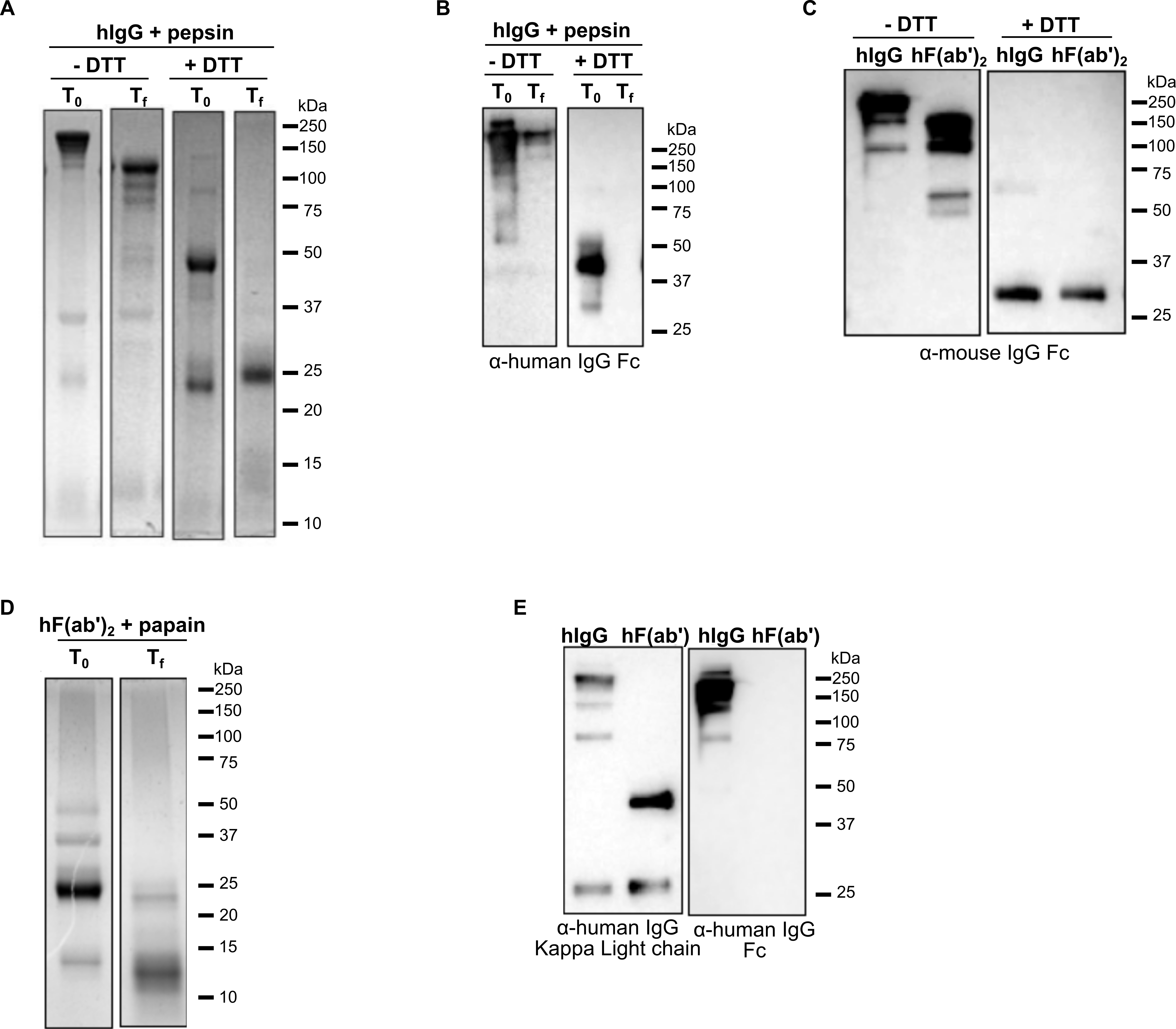

Supplement: FIG S6 [file mbio.03059-21-sf006.tif]

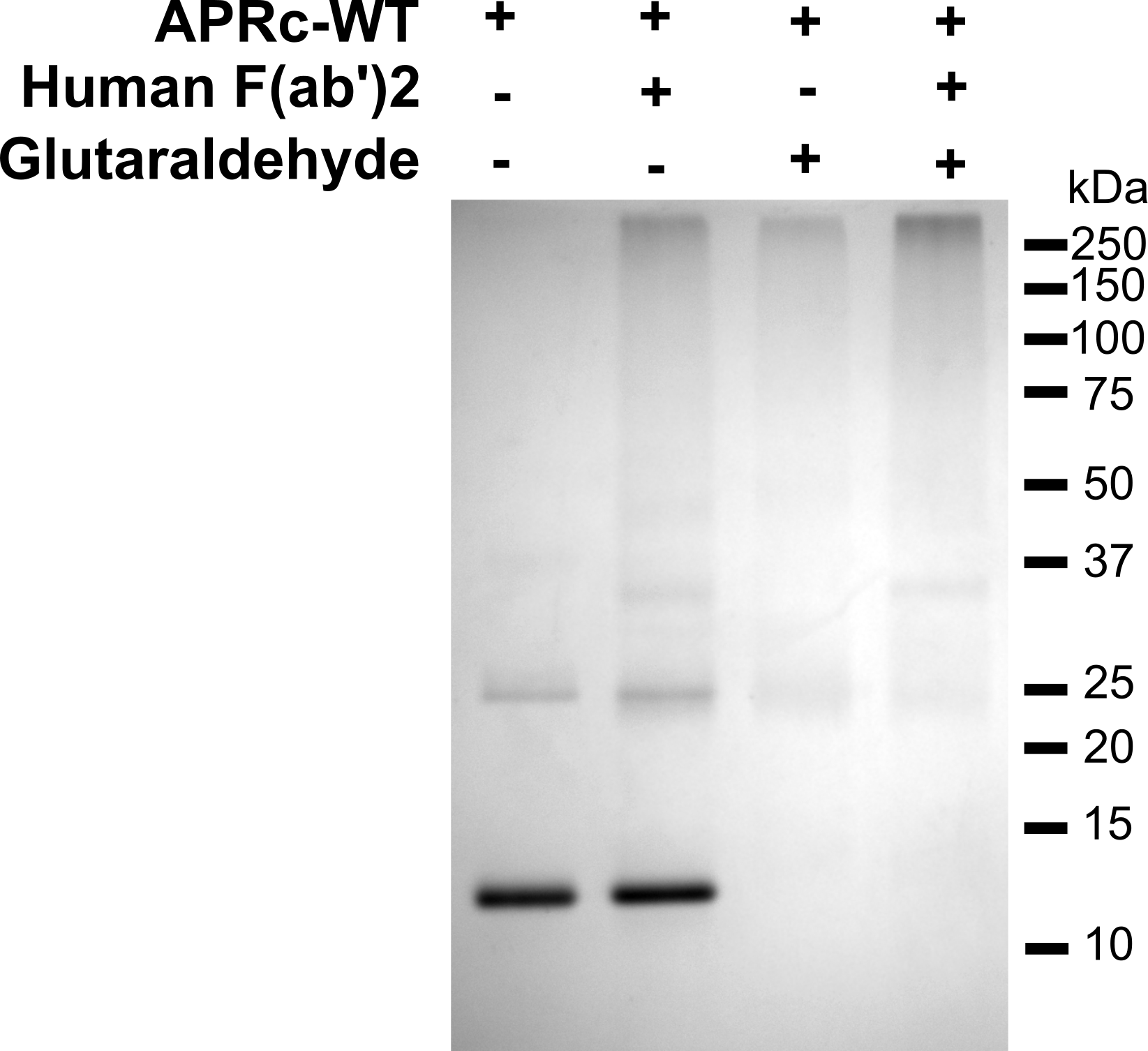

Supplement: FIG S7 [file mbio.03059-21-sf007.tif]

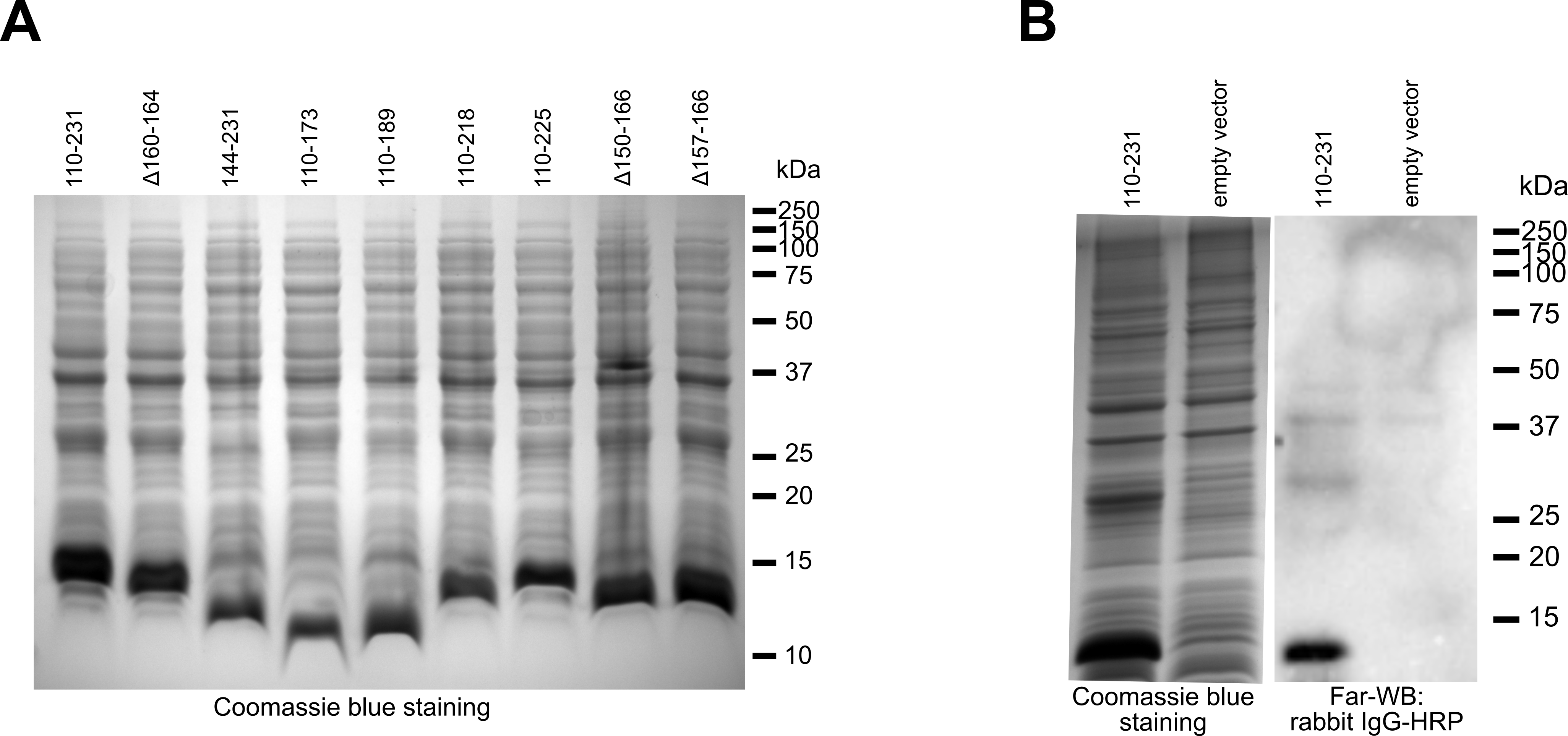

Supplement: FIG S8 [file mbio.03059-21-sf008.tif]

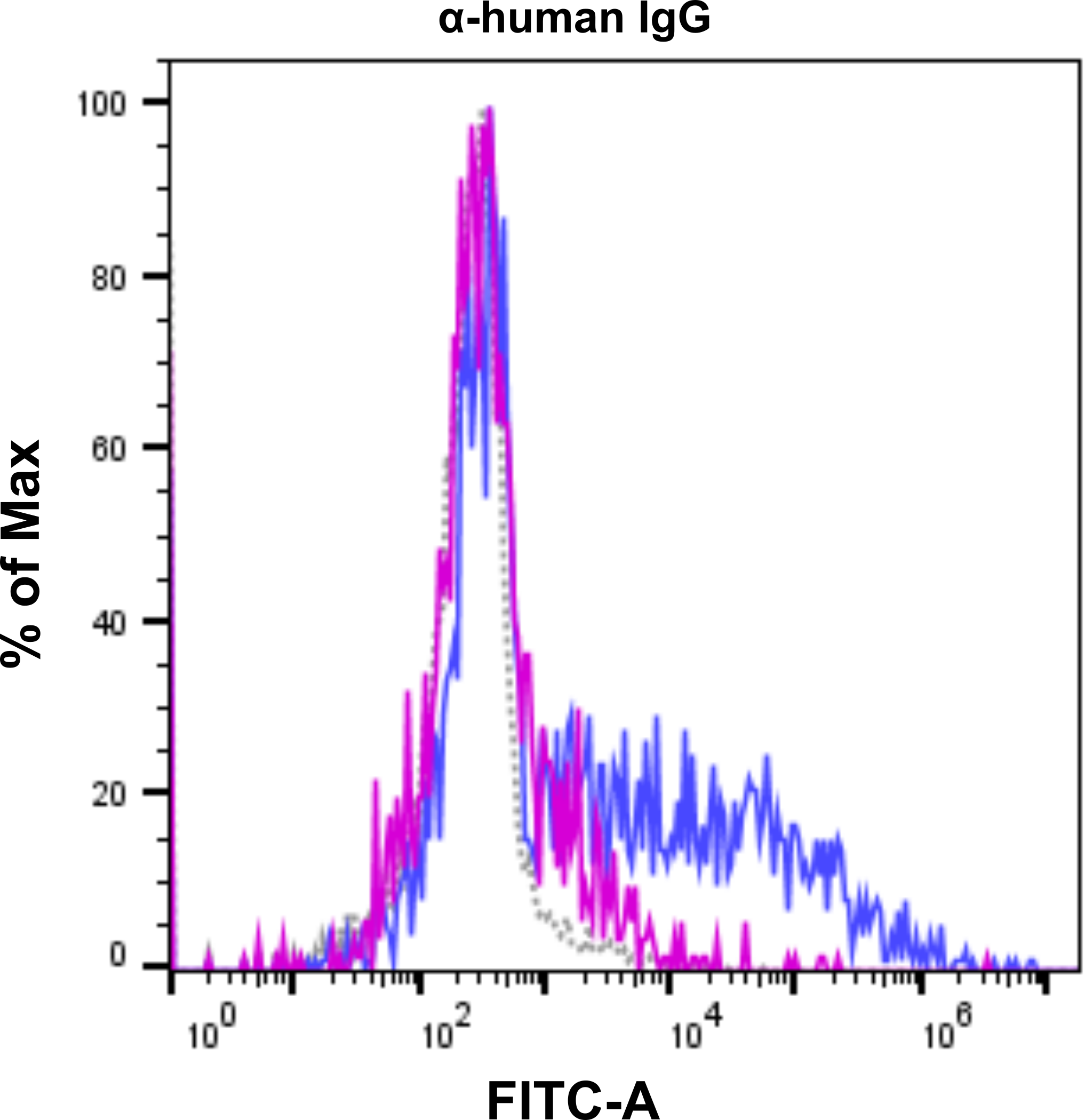

Supplement: FIG S9 [file mbio.03059-21-sf009.tif]
