# Supplementary material for: The Retropepsin-Type Protease APRc as a Novel Ig-Binding Protein and Moonlighting Immune Evasion Factor of Rickettsia
Source: mBio. 2021 Dec 7;12(6):e03059-21. doi: 10.1128/mBio.03059-21 (PMC8649778; doi:10.1128/mBio.03059-21)
Supplement: TABLE S1 [file mbio.03059-21-st001.pdf]

Table S1. DNA constructs used in this study

DNA construct	***Primers***
pEThttps://www.sharedfilespro.com/shared-files/38/?APRc$_{144\text{-}231}$HisShort	forward primer:<br>5’-GCCTCTGATATTGCACTGAC-3’<br>reverse primer:<br>5’-CATATGTATATCTCCTTCTTAAAGTTAAACAAA-3’
pET_APRc(Δ160-164)$_{110\text{-}231}$HisShort	forward primer:<br>5’-TATACCCGTACGTACCTGACG-3’<br>reverse primer:<br>5’-ATC GAAACCCAGTTTCTGC-3’
pET_APRc(Δ157-166)$_{110\text{-}231}$HisShort	forward primer:<br>5’-CGTACGTACCTGACGGCC-3’<br>reverse primer:<br>5’-CAGTTTCTGCGCATCTTCTT-3’
pET_APRc(Δ150-166)$_{110\text{-}231}$HisShort	forward primer:<br>5’- CGTACGTACCTGACGGCC-3’<br>reverse primer:<br>5’-CGTCAGTGCAATATCAGAGG-3’
pET_APRc$_{110\text{-}173}$HisShort	forward primer:<br>5’-CACCACCACCACCACCAC-3’<br>reverse primer:<br>5’-GTTGGCCGTCAG GTACGT-3’
pET_APRc$_{110\text{-}189}$HisShort	forward primer:<br>5’-CACCACCACCACCACCAC -3’<br>reverse primer:<br>5’-GCCGATAACCAC GCTGTT-3’
pET_APRc$_{110\text{-}218}$HisShort	forward primer:<br>5’-CACCACCACCACCACCAC-3’<br>reverse primer:<br>5’-TTTAAAACGTTC CAGCAGAGAC-3’
pET_APRc$_{110\text{-}225}$HisShort	forward primer:<br>5’-CACCACCACCACCACCAC-3’<br>reverse primer:<br>5’-ATC TTTATCGATGCGGAAACC-3’
pCoofy_HisAPRc$_{110\text{-}231}$-avi	forward primer:<br>5’GGATCCGGACTGAACGACATCTTC-3’<br>reverse primer:<br>5’-GGGCCCCTGGAACAGAACTTC-3’<br>forward primer:<br>5’AAGTTCTGTTCCAGGGGCCCGAAGTTGGCGAAATTATCATT-3’<br>reverse primer:<br>5’GAAGATGTCGTTCAGTCCGGATCCATAATTCAGAATCAGCAGATCTTTAT-3’
